# Epigenomic landscapes define differential Janus kinases inhibitor sensitivity in IFN-γ-primed human macrophages

**DOI:** 10.1016/j.isci.2025.112502

**Published:** 2025-04-22

**Authors:** Geunho Kwon, Yebin Park, Keunsoo Kang, Kyung-Hyun Park-Min, Kyuho Kang

**Affiliations:** 1 Department of Biological Sciences and Biotechnology, Chungbuk National University, Cheongju 28644, Republic of Korea; 2 Department of Microbiology, College of Science & Technology, Dankook University, Cheonan 31116, Republic of Korea; 3 Arthritis and Tissue Degeneration Program, David Z. Rosensweig Genomics Research Center, Hospital for Special Surgery, New York, NY 10021, USA; 4 BCMB Allied Program, Weill Cornell Graduate School of Medical Sciences, New York, NY 10021, USA; 5 Department of Medicine, Weill Cornell Medical College, New York, NY 10065, USA

**Keywords:** Components of the immune system, Immunology, Transcriptomics

## Abstract

Janus kinases (JAK) mediate signaling pathways of multiple cytokines, including interferon-γ (IFN-γ), which plays a pivotal role in rheumatoid arthritis (RA) pathogenesis. Although JAK inhibitors (JAKi) have demonstrated efficacy for RA, their molecular effects on macrophages remain incompletely understood. We investigate the impact of JAKi on IFN-γ-induced gene expression in human macrophages, uncovering that JAKi selectively modulates only a subset of IFN-γ-induced genes. Integrated transcriptomic and epigenomic analyses demonstrate that JAKi effectively inhibits IFN-γ signature genes associated with IRF1-STAT1-dependent accessible chromatin regions. However, genes regulated by AP-1 and C/EBP remain insensitive to JAKi and overlap significantly with TNF-induced genes. Single-cell analysis of RA patient samples identifies macrophage subpopulations with variable JAKi sensitivity. Certain JAKi-insensitive genes in IFN-γ-primed macrophages are suppressed by JNK inhibitors. Our findings elucidate JAKi responsiveness mechanisms through IFN-γ-induced epigenomic remodeling, providing insights into inflammatory regulation in RA and suggesting strategies to overcome JAKi resistance.

## Highlights

• JAKi selectively inhibits IFN-γ signature genes associated with IRF1-STAT1-dependent ACRs
• Genes with AP-1 and C/EBP motifs are JAKi-insensitive and overlap with TNF-induced genes
• ScRNA-seq reveals heterogeneous JAK inhibitor responsiveness in RA macrophage subpopulations
• JNK inhibitors suppress certain JAKi-insensitive genes in IFN-γ-primed macrophages

## Graphical abstract

**Figure:**
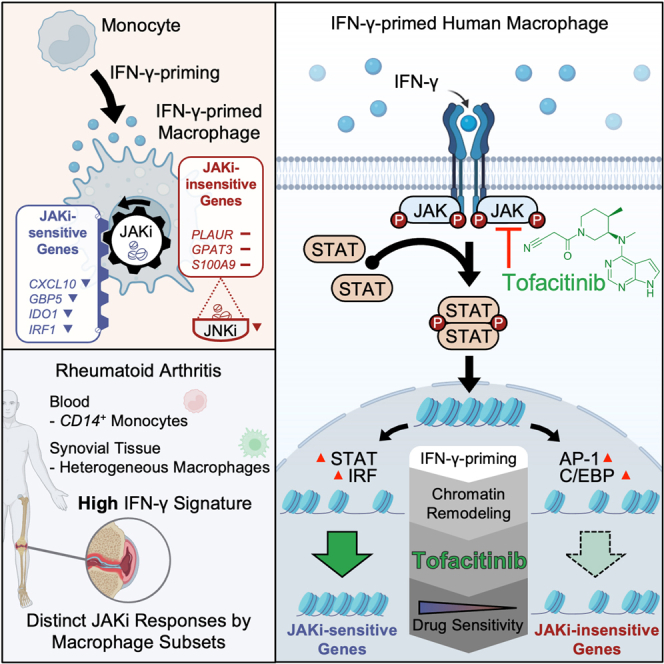

## Introduction

Inflammation is a multifaceted immune response triggered by various stimuli, such as pathogens and damaged cells.^1^ Dysregulated inflammation underlies the pathogenesis of numerous autoimmune and inflammatory diseases, including systemic lupus erythematosus (SLE), rheumatoid arthritis (RA), inflammatory bowel disease (IBD), and COVID-19.^2^^,^^3^^,^^4^ These conditions are characterized by abnormal immune responses and excessive production of pro-inflammatory cytokines, particularly TNF and IL-6.^5^^,^^6^^,^^7^

Macrophages are central to this response, exhibiting dynamic changes in their transcriptomic, epigenomic, and metabolic programs in response to various signals.^8^^,^^9^^,^^10^ These cells display diverse phenotypes and functions shaped by signals from the tissue microenvironment.^3^^,^^11^^,^^12^^,^^13^^,^^14^^,^^15^ A hallmark of many inflammatory diseases is the presence of an “interferon (IFN) signature” in innate immune cells, including macrophages.^4^^,^^16^^,^^17^ Pathogenic macrophages in inflammatory diseases are activated by the combined effects of IFN-γ and other cytokines.^4^^,^^18^ Gaining a deeper understanding of the molecular mechanisms associated with IFN signature is crucial for comprehending the underlying mechanisms of inflammatory diseases.

IFN-γ is a key pro-inflammatory cytokine that drives macrophage activation toward a pro-inflammatory “M1-like” phenotype.^16^ IFN-γ signaling is mediated through the JAK-STAT pathway, primarily activating JAK1 and JAK2.^19^ This activation leads to the phosphorylation of both STAT1 and STAT3, which compete for binding to specific *cis*-regulatory elements (CRE), influencing gene expression and inflammatory responses.^20^^,^^21^^,^^22^^,^^23^ IFN-γ priming induces epigenetic modifications and metabolic reprogramming in macrophages, resulting in enhanced expression of pro-inflammatory genes and suppression of anti-inflammatory genes.^24^^,^^25^ This “epigenomic signature” is associated with the development and progression of inflammatory diseases.^26^^,^^27^ A deeper understanding of the interactions between these signaling pathways and their epigenetic regulation by STATs and other transcription factors (TF) in the context of inflammatory diseases is crucial for the development of effective therapies.

The JAK-STAT pathway has emerged as a promising therapeutic target for inflammatory diseases. JAK inhibitors (JAKi) have demonstrated efficacy in treating conditions such as RA by modulating immune responses and suppressing pro-inflammatory cytokine production.^28^^,^^29^ The shared transcriptomic profiles, including IFN signatures, observed across various inflammatory diseases, suggest the potential for a common therapeutic approach using JAKi.^4^ Tofacitinib, an oral pan-JAKi, exemplifies this therapeutic strategy by effectively blocking the activity of JAK1, JAK3, and partially JAK2. This inhibition leads to a reduction in pro-inflammatory cytokine production and promotes a more balanced immune response.^30^ In clinical practice, tofacitinib is often used in combination with other disease-modifying anti-rheumatic drugs (DMARDs) or as a last-line treatment for patients refractory to conventional therapies, such as methotrexate (MTX) and TNF inhibitors.^31^ Recent studies have highlighted the importance of JAKi treatment in modulating the epigenetic landscape of immune cells. Repeated JAKi treatment has been demonstrated to repress IFN-signature genes and alter chromatin accessibility at CRE.^32^^,^^33^ These findings underscore the complex interplay between JAK-STAT signaling and epigenetic regulation in inflammatory responses. Recent research has suggested their utility in managing cytokine storms associated with severe COVID-19, highlighting the broader implications of JAK-STAT pathway modulation in immune regulation.^34^^,^^35^ Despite their therapeutic promise, safety concerns regarding JAKi, particularly with regard to cardiovascular and cancer risk, have been raised in patients with older RA with cardiovascular risk factors.^36^^,^^37^ To fully harness the potential of JAKi in treating inflammatory diseases such as RA and potentially expand their therapeutic applications, a deeper understanding of the molecular mechanisms underlying their regulation of inflammatory responses is necessary.

In this study, we investigated the molecular mechanisms underlying the JAKi regulation of IFN-γ-induced gene expression in human macrophages. Through integrated transcriptomic and epigenomic analyses, we identified distinct subsets of IFN-γ signature genes characterized by differential sensitivity to JAK inhibition. JAKi treatment decreased IFN-γ-induced phosphorylation of both STAT1 and STAT3, which led to reduced TF occupancy and chromatin accessibility at CRE containing IRF-STAT binding motifs. However, genes that remained insensitive to JAK inhibition maintained their accessible chromatin regions (ACR), which were characterized by enriched binding motifs for AP-1 and C/EBP. To explore the clinical relevance of these findings, we examined macrophage subpopulations in RA synovial tissue, identifying distinct transcriptomic and epigenetic signatures associated with differential JAKi sensitivity. Notably, c-Jun N-terminal kinase (JNK) inhibition suppressed some JAKi-insensitive genes in IFN-γ-primed macrophages. These findings elucidate epigenomic mechanisms of JAKi responsiveness in IFN-γ-primed human macrophages and suggest strategies for targeting resilient gene sets in inflammatory diseases.

## Results

### Janus kinases inhibitor selectively regulates interferon-γ-induced genes in macrophages

**Figure 1:**
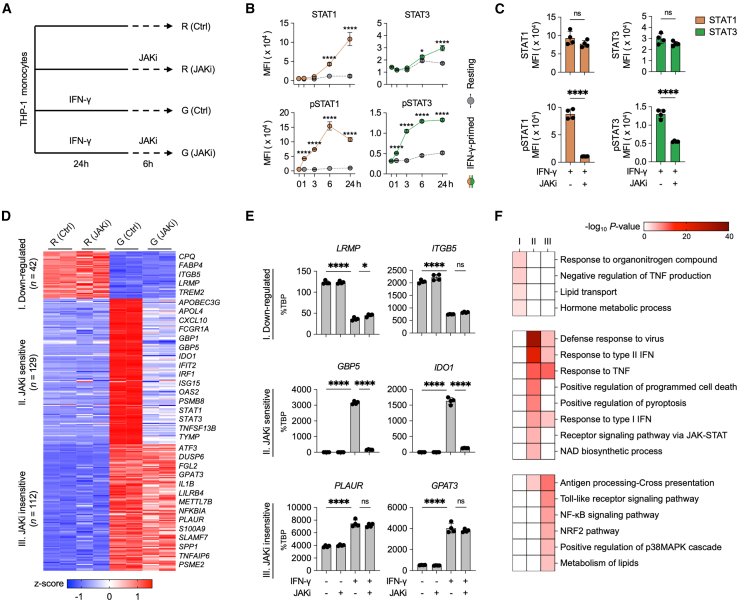
JAK inhibition differentially regulates IFN-γ-inducible gene expression in macrophages (A) Schematic representation of the experimental design. THP-1 cells were differentiated into macrophages using PMA (100 nM) for 24 h, primed with IFN-γ (100 U/ml) or left unprimed for 24 h, and subsequently treated with JAKi (tofacitinib) or vehicle (DMSO) for 6 h. (B) Flow cytometric analysis of total and phosphorylated STAT1 (left) and STAT3 (right) levels in THP-1-derived macrophages treated with PMA and IFN-γ (or without) at various time points. Mean fluorescence intensity (MFI) values are shown. Three independent experiments were performed (*n* = 3). (C) Flow cytometric analysis of total and phosphorylated STAT1 (left) and STAT3 (right) levels in THP-1-derived macrophages treated as described in Figure 1A. MFI values were measured after 6 h of JAKi treatment. Four independent experiments were performed (*n* = 4). (D) *k*-means clustering of DEG identified by edgeR (FDR-adjusted *p* < 0.05, |Fold change| > 2) in THP-1-derived macrophages from pairwise comparisons between the four experimental conditions. DEGs were cross-validated against HMDM transcriptome data (GEO: GSE98368) through positive correlation analysis. The color scale represents z-scores. (E) RT-qPCR analysis of target mRNA levels in THP-1-derived macrophages under the indicated conditions, normalized to TATA binding protein (TBP) mRNA. Two independent experiments were performed. (F) Gene ontology (GO) analysis using DEG from THP-1-derived macrophages. The heatmap displays the significance (-log_10_*p*-value) of GO term enrichment for genes in each cluster: I (*n* = 42), II (*n* = 129), and III (*n* = 112). GO analysis was performed using Metascape (http://metascape.org/). Data are presented as mean ± standard deviation (SD), with statistical significance determined by one-way analysis of variance (ANOVA) followed by Tukey’s multiple comparisons test (ns, not significant; ∗*p* < 0.05; ∗∗*p* < 0.01; ∗∗∗*p* < 0.001; ∗∗∗∗*p* < 0.0001).

To elucidate the molecular mechanisms underlying IFN-γ-mediated effects and JAK inhibitor (JAKi) regulation in macrophages, we established four experimental conditions (Figure 1A). IFN-γ priming for 24 h significantly increased the levels of both total and phosphorylated STAT1 and STAT3 compared to resting macrophages (Figure 1B). STAT1 and STAT3 phosphorylation were rapidly induced at early time points following IFN-γ stimulation. We next examined the JAKi-mediated modulation of gene expression in IFN-γ-primed macrophages. JAKi dose-dependently suppressed IFN-γ-JAK-STAT axis target genes (*CCL2* and *CXCL10*), with maximal inhibition at 1 μM (Figure S1A). JAKi treatment markedly reduced STAT1 and STAT3 phosphorylation while minimally affecting their total protein levels in IFN-γ-primed macrophages (Figure 1C). To assess the temporal dynamics of JAKi-mediated inhibition, we monitored the expression of IFN-γ-induced genes. JAKi suppressed *CCL2* and *CXCL10* expression in a time-dependent manner, with maximal inhibition observed at 6 h post-treatment (Figure S1B).

To comprehensively assess the impact of JAKi on IFN-γ-primed macrophages, we conducted RNA sequencing (RNA-seq) analysis. We identified 283 differentially expressed genes (DEG) through pairwise comparisons among the four conditions. JAKi induced more substantial transcriptomic changes in IFN-γ-primed macrophages compared to resting macrophages (Figure S1C). We further classified the DEG into three clusters based on *k*-means clustering (Figure 1D). Cluster I (42 genes) contained IFN-γ-suppressed genes that remained unaffected by JAKi (Figures 1D and 1E). Notably, IFN-γ-induced genes segregated into two clusters based on JAKi sensitivity: cluster II (129 JAKi-sensitive genes) and cluster III (112 JAKi-insensitive genes). Cluster II genes, including *GBP5* and *IDO1*, were associated with IFN signaling and JAK-STAT pathways (Figures 1E and 1F). In contrast, cluster III genes, such as *PLAUR* and *GPAT3*, were linked to antigen presentation, lipid metabolism, and mitogen-activated protein kinase (MAPK) cascade-related functions. These findings demonstrate that JAKi efficiently inhibit the JAK-STAT pathway in IFN-γ-primed macrophages, with distinct gene clusters exhibiting differential sensitivity to JAK inhibition. This selective regulation suggests the involvement of complex molecular mechanisms beyond the straightforward inhibition of the JAK-STAT pathway.

To investigate whether phorbol 12-myristate 13-acetate (PMA)-mediated differentiation influences macrophage activation,^38^ we conducted a systematic analysis of PMA dose-dependent effects. Our examination of differentiation markers revealed distinct cellular responses across PMA concentrations. At low concentrations (0–1 nM), PMA treatment maintained elevated expression of the monocyte marker *CCR2* while inducing the minimal expression of macrophage-specific markers *FCGR1A* and *CD68*. In contrast, treatment with higher PMA concentrations (10–100 nM) effectively promoted macrophage differentiation (Figure S1D). Importantly, we observed that the expression levels of genes associated with NF-κB signaling, stress response pathways, and IFN responsiveness remained consistent across all tested PMA concentrations (Figures S1E–S1G). These findings indicate that PMA-induced differentiation does not significantly alter the IFN-γ priming response in macrophages.

### Differential open chromatin changes by Janus kinases inhibitors in interferon-γ-primed macrophages

**Figure 2:**
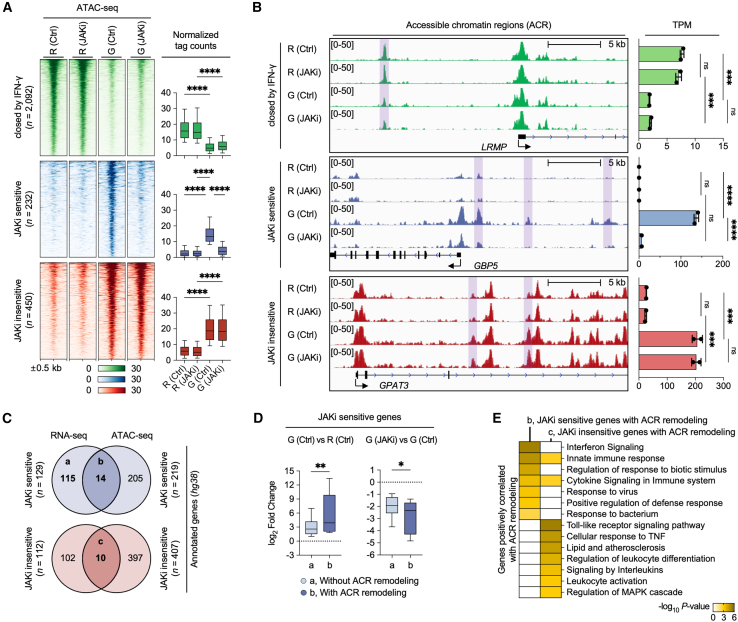
JAK inhibition differentially regulates chromatin accessibility and gene expression in THP-1-derived human macrophages (A) Heatmap of ATAC-seq signals in THP-1-derived macrophages treated as described in (Figure 1A) (left). Differentially regulated ACRs were identified by edgeR (FDR-adjusted *p* < 0.05, |Fold change| > 2). Boxplots show normalized tag counts for ATAC-seq peaks (right). ATAC-seq peaks were cross-validated against HMDM ATAC-seq data (GEO: GSE98365) through positive correlation analysis. (B) Example of ATAC-seq signals in a selected region from the heatmap cluster. IGV tracks display ATAC-seq signals near the indicated genes in THP-1-derived macrophages (left). Bar graphs show the expression levels of the indicated genes (right). (C) Venn diagrams for identifying positively correlated genes in RNA-seq and ATAC-seq. For ATAC-seq analysis, an annotated gene in the *hg38* genome was used. (D) Boxplots show the fold change difference for genes with and without chromatin remodeling. JAKi-sensitive gene sets “a” and “b,” identified by ATAC-seq in (Figure 2C), were used. The fold change in gene expression between the macrophage conditions indicated above the boxplot was used. (E) GO analysis using genes identified in the integrated analysis of RNA-seq and ATAC-seq from THP-1-derived macrophages. The heatmap shows the significance (-log_10_*p*-value) of GO term enrichment for each gene set (b, JAKi-sensitive, *n* = 20; c, JAKi-insensitive, *n* = 136). Boxes, 25th-75th percentile; whiskers, 10th and 90th percentiles; central horizontal bar, median. Data are presented as mean ± SD, with statistical significance determined by one-way ANOVA followed by Tukey’s multiple comparisons test (ns, not significant; ∗*p* < 0.05; ∗∗*p* < 0.01; ∗∗∗*p* < 0.001; ∗∗∗∗*p* < 0.0001). GO analysis was performed using Metascape.

To gain insight into the epigenetic mechanisms underlying differential gene sensitivity to JAK inhibition, we performed Assay for Transposase-Accessible Chromatin with sequencing (ATAC-seq) under conditions identical to our RNA-seq analysis (Figure 1A). Sample quality was assessed based on fragment size distribution (Figure S2A). We identified 2,774 differentially ACR (FDR <0.05, |Fold change| > 2) across pairwise comparisons of the four conditions. Principal component analysis revealed that JAK inhibition elicited more extensive chromatin reorganization in IFN-γ-primed macrophages compared to resting macrophages, suggesting that the chromatin landscape’s response to JAK inhibition is significantly influenced by the prior activation state of the macrophages (Figure S2B). We identified three major ACR clusters (Figures 2A and 2B). The first cluster, comprising 2,092 ACR closed by IFN-γ, showed decreased accessibility independent of JAKi, as demonstrated by the promoter and enhancers of the representative gene *LRMP*. The second cluster, termed “JAKi-sensitive” (*n* = 232), consisted of open chromatin regions induced by IFN-γ but significantly inhibited by JAKi, exemplified by the *cis*-regulatory regions of *GBP5*. In contrast, the “JAKi-insensitive” cluster (*n* = 450) contained open chromatin regions induced by IFN-γ that were unaffected by JAKi, as demonstrated in intronic enhancers of the *GPAT3* gene.

Integrated analysis of RNA-seq and ATAC-seq data revealed that 14 of 129 JAKi-sensitive genes overlapped with annotated genes in the JAKi-sensitive ACR cluster, while 10 of 112 JAKi-insensitive genes overlapped with annotated genes in the JAKi-insensitive ACR cluster (Figure 2C). Genes with ACR remodeling exhibited greater expression changes in response to IFN-γ and JAKi compared to genes without ACR remodeling (Figure 2D). Functional gene ontology (GO) analysis revealed that JAKi-sensitive genes with ACR remodeling were enriched for IFN signaling and antiviral response pathways (Figures 2E and S2C). Conversely, JAKi-insensitive genes with ACR remodeling were associated with Toll-like receptor 4 (TLR4) signaling, cellular response to TNF, and MAPK cascade functions. Our findings reveal that IFN-γ-primed macrophages contain distinct classes of ACR characterized by their differential sensitivity to JAK inhibition, indicating that this selective chromatin accessibility serves as a crucial mechanism for regulating specific subsets of IFN-γ-induced genes.

### Distinct transcription factor repertoires in Janus kinases inhibitors-regulated genes

**Figure 3:**
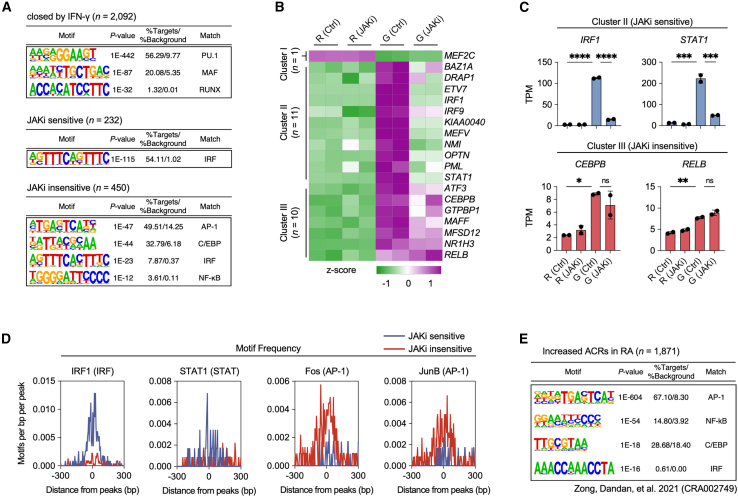
JAK inhibition differentially modulates TF expression and motif enrichment in THP-1-derived human macrophages (A) *De novo* motif analysis using HOMER identified the most significantly enriched TF motifs in each cluster of peaks. The enriched motifs for each cluster are shown. (B) Heatmap of gene expression for 94 TF in clusters defined by RNA-seq data analysis of THP-1-monocyte-derived IFN-γ-primed macrophages. (C) Bar plots show the expression levels of representative TF from the JAKi-sensitive (*IRF1* and *STAT1*) and JAKi-insensitive (*CEBPB* and *RELB*) clusters identified in the heatmap of THP-1-derived macrophages. (D) Motif frequency plots show the distribution of the enriched motifs (IRF1, STAT1, Fos, and JunB) relative to the peak center in the JAKi-sensitive and -insensitive clusters. (E) *De novo* motif analysis of regions of increased chromatin accessibility (*n* = 1,871) in RA monocytes compared to OA monocytes. The most abundant motifs are shown for regions of increased chromatin accessibility in patients with RA. Data are presented as mean ± SD, with statistical significance determined by one-way ANOVA followed by Tukey’s multiple comparisons test (ns, not significant; ∗*p* < 0.05; ∗∗*p* < 0.01; ∗∗∗*p* < 0.001; ∗∗∗∗*p* < 0.0001).

To focus on the regulatory mechanisms underlying JAKi sensitivity in IFN-γ-primed macrophages, we investigated the associations of transcription factors (TF) with identified ACR. Through *de novo* motif analysis, we uncovered distinct patterns of motif enrichment across ACR clusters (Figure 3A). In the cluster of ACR that showed reduced accessibility following IFN-γ treatment, we identified significant enrichment of PU.1 and MAF motifs. The presence of MAF motifs in these regions is consistent with previous research demonstrating that IFN-γ suppresses gene expression through the disruption of MAF-bound enhancers.^25^ The enrichment of PU.1 motifs in these regions is particularly noteworthy, as PU.1 is known to designate enhancer regions for TF binding in macrophages.^39^^,^^40^ The observed decrease in chromatin accessibility within these regions after IFN-γ treatment suggests the selective regulation of chromatin states at PU.1-marked enhancers during inflammatory responses. Importantly, JAK inhibition did not affect the chromatin accessibility changes in these regions. JAKi-sensitive ACR showed significant enrichment of IRF motifs, aligning with previous findings on STAT1 and IRF1 in IFN-γ-primed macrophages.^41^ In contrast, the JAKi-insensitive ACR cluster showed pronounced enrichment of AP-1 motifs, supporting our earlier observation of enhanced "p38MAPK cascade" function in the JAKi-insensitive gene cluster (Figure 1).

We next examined the expression levels of differentially expressed TF across the three identified clusters (Figure 3B). JAKi-sensitive TF (*n* = 11), including *IRF1*, *IRF9*, and *STAT1*, were repressed by JAKi in IFN-γ-primed macrophages (Figures 3B and 3C). In contrast, JAKi-insensitive TF (*n* = 10), such as *CEBPB*, and *RELB*, remained unaffected by JAKi treatment. To further understand the relationship between JAK inhibition and IRF-mediated responses, we conducted additional analyses based on previous evidence that JAK-STAT and IRF3-mediated pathways can function independently during macrophage activation.^42^ Although IRF motifs showed enrichment in JAKi-sensitive regions, we also detected their presence in JAKi-insensitive regions (Figure 3A). Notably, in IFN-γ-primed macrophages, the expression of *IRF3* remained constant despite JAK inhibition (Figures S3A and S3B), indicating that different members of the IRF family are subject to distinct regulatory mechanisms. To validate the *de novo* motif analysis results, we investigated known motifs for JAKi-sensitive and JAKi-insensitive ACR, revealing distinct patterns of PU.1, IRF, and AP-1 motifs for each ACR cluster (Figure S3C). Motif frequency analysis further corroborated these findings, with IRF family motifs, including IRF1, more frequently observed in JAKi-sensitive ACR (Figure 3D). Conversely, AP-1 family motifs, such as Fos and JunB, were significantly more prevalent in JAKi-insensitive ACR. Interestingly, motif analysis of public ATAC-seq data (NGDC: CRA002749) from RA patient monocytes showed enrichment of AP-1, NF-κB, C/EBP, and IRF motifs in differential ACR compared to patients with osteoarthritis (OA) (Figure 3E).^43^ This suggests that both JAKi-sensitive and JAKi-insensitive ACR might be involved in open chromatin regions in RA monocytes. These results reveal distinct TF repertoires associated with JAKi sensitivity in IFN-γ-primed macrophages. The preferential enrichment of IRF motifs in JAKi-sensitive regions and AP-1 motifs in JAKi-insensitive regions provides insight into the molecular basis of the observed transcriptional heterogeneity in response to JAK inhibition.

### Cytokine-specific gene signatures reveal differential Janus kinases inhibitor sensitivity

**Figure 4:**
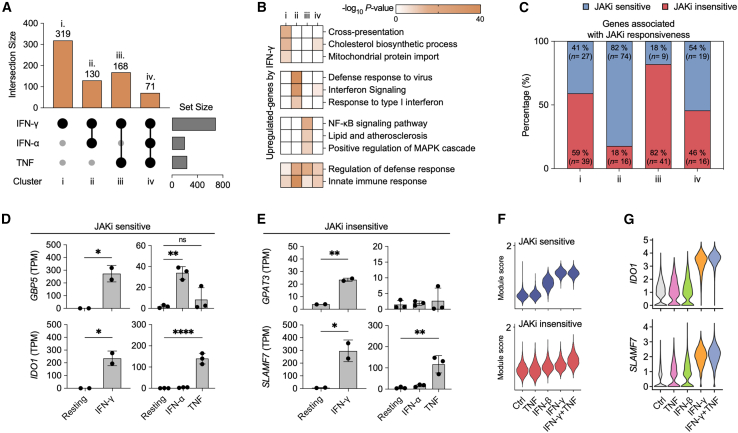
JAK inhibition differentially modulates IFN-γ, IFN-α, and TNF-induced gene expression in human monocyte-derived macrophages (HMDM) (A) UpSet plot depicts the magnitude of the interaction between IFN-γ, IFN-α, and TNF stimulation across gene sets (clusters i-iv) in HMDM. Numbers above intersections indicate the size of shared and unique gene sets. DEG were identified by edgeR (FDR-adjusted *p* < 0.05, |Fold change| > 2). (B) GO enrichment analysis shows the top biological pathways/processes corresponding to each cluster. Color intensity represents the *p*-value (-log_10_). (C) Distribution of JAKi-sensitive (blue) and JAKi-insensitive (red) genes identified from THP-1 analysis across clusters. (D and E) Expression of JAKi-sensitive genes (*GBP5* and *IDO1*) and JAKi-insensitive genes (*GPAT3* and *SLAMF7*) in HMDM, using gene sets defined from THP-1 analysis. (F) Module scores of JAKi-sensitive and -insensitive gene sets defined from THP-1 analysis across different stimuli in HMDM. (G) Expression profiles of representative genes (*IDO1* and *SLAMF7*) under different conditions for clusters ii and iii in HMDM. Data are presented as mean ± SD, with statistical significance determined by one-way ANOVA followed by Tukey’s multiple comparisons test (ns, not significant; ∗*p* < 0.05; ∗∗*p* < 0.01; ∗∗∗*p* < 0.001; ∗∗∗∗*p* < 0.0001). GO analysis was performed using Metascape.

To delineate the unique transcriptional profiles induced by different inflammatory stimuli, we compared the gene expression profiles of IFN-γ-primed macrophages to those stimulated by IFN-α and TNF (GEO: GSE98368 and GEO: GSE100382) (Figures 4A and S4A).^6^^,^^25^ Upregulated genes in IFN-γ-primed macrophages were classified into four clusters: IFN-γ-specific (Cluster i, *n* = 319), IFN-α-shared (Cluster ii, *n* = 130), TNF-shared (Cluster iii, *n* = 168), and genes shared by all three cytokine stimuli (Cluster iv, *n* = 71). GO analysis revealed unique functional associations for each cluster (Figure 4B). Cluster i genes were enriched for “cross-presentation” and “cholesterol biosynthetic process,” Cluster ii for “antiviral defense responses,” and Cluster iii for “NF-κB signaling pathway” and “MAPK cascade.” Notably, Cluster ii had a higher proportion of JAKi-sensitive genes, such as *GBP5* and *IDO1*, while Cluster iii contained more JAKi-insensitive genes, including *GPAT3* and *SLAMF7* (Figures 4C–4E). Analysis of TF identified by *de novo* motif analysis confirmed their cytokine-specific regulation. *IRF1* was upregulated by both IFN-α and TNF, *STAT1* was specifically regulated by IFN-α, and *CEBPB* and *RELB* were specifically regulated by TNF (Figures S4B and S4C). This pattern suggests that the shared gene signatures between IFN-γ and IFN-α are more likely to be JAK-dependent, while those shared with TNF show greater resistance to JAK inhibition.

To explore cytokine-induced transcriptional heterogeneity at the single-cell level, we analyzed single-cell RNA sequencing (scRNA-seq) data (GEO: GSE168710) from human monocyte-derived macrophages (HMDM) (Figures S4D and S4E).^4^ We focused on three key conditions: macrophages co-stimulated with IFN-γ and TNF, which have been reported to induce a *CXCL10*^+^ *CCL2*^+^ phenotype, and macrophages mono-stimulated with either IFN-γ or IFN-β.^4^ Our analysis revealed distinct expression patterns of JAKi-sensitive and JAKi-insensitive gene modules across these conditions (Figure 4F). The JAKi-sensitive module showed high expression in both IFN-γ- and IFN-β-stimulated HMDM, suggesting a common type I and type II IFN-induced signature. In contrast, the JAKi-insensitive module was predominantly expressed in IFN-γ and TNF co-stimulated macrophages, indicating a synergistic effect of these cytokines on this gene set. The expression of JAKi-sensitive genes, such as *IDO1*, reached peak levels in response to IFN-γ stimulation regardless of TNF presence. In contrast, JAKi-insensitive genes, including *SLAMF7*, displayed a synergistic response pattern, with expression levels reaching their maximum specifically under dual stimulation with IFN-γ and TNF in HMDM (Figure 4G).

While neither IFN-α nor TNF alone affected *TNF* expression in primary HMDM, IFN-γ priming induced a modest but significant upregulation that was further enhanced under IFN-γ and TNF co-stimulation (Figures S4F and S4G). AP-1 family members, including *FOS* and *JUNB*, which were identified as top *de novo* motifs in JAKi-insensitive chromatin regions (Figures 3A and S3C), showed increased expression in response to TNF stimulation (Figure S4H). ChIP sequencing (ChIP-seq) analysis revealed increased NF-κB binding upon IFN-γ priming in both JAKi-sensitive and -insensitive genes. However, the chromatin remodeling region identified in the *GPAT3* loci, a JAKi-insensitive gene, was found to be independent of NF-κB signaling (Figure S4I). This finding suggests that while NF-κB signaling contributes to IFN-γ-responsive gene regulation, chromatin remodeling at specific JAKi-insensitive gene loci may proceed through NF-κB-independent mechanisms. Unbiased gene expression analysis demonstrated a significant increase in both JAKi-sensitive and -insensitive genes in IFN-γ- and TNF-co-stimulated macrophages (Figures S5A–S5C), indicating a unique transcriptional landscape associated with specific IFN-γ signatures in human macrophages. These findings highlight the differential regulation of IFN-γ signature genes through JAK activation and reveal distinct yet overlapping functional roles with genes regulated by IFN-α and TNF signaling pathways.

### Janus kinases inhibitors selectively suppress STAT1 and IRF1 binding to *cis*-regulatory regions

**Figure 5:**
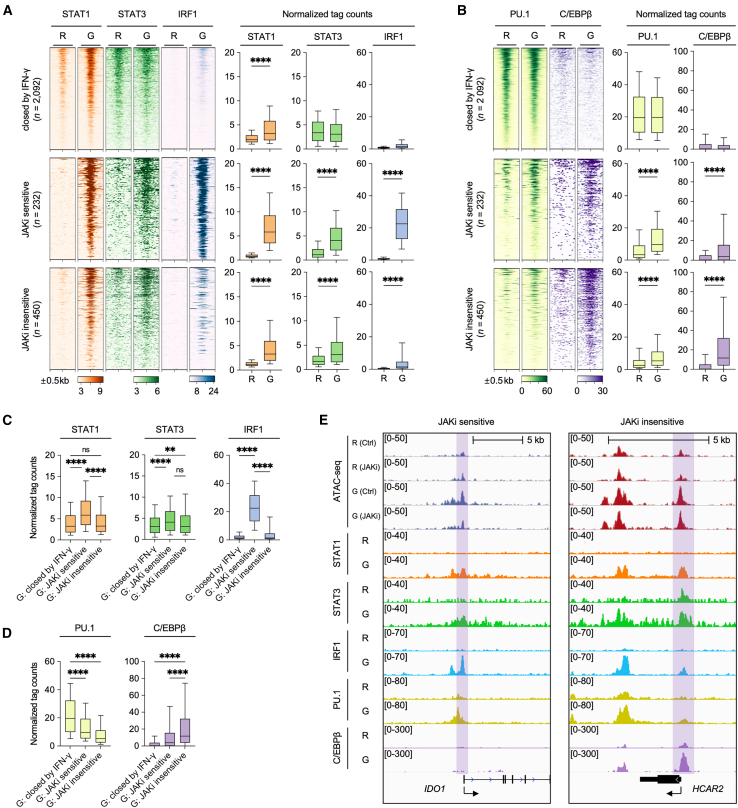
Distinct TF binds to ACR regulated by JAKi (A and B) Heatmap of ChIP-seq (STAT1, STAT3, IRF1 PU.1 and C/EBPβ) signals in HMDM with or without IFN-γ-priming (left). ChIP-seq data from HMDM were obtained from GEO: GSE43036, GEO: GSE120943, and GEO: GSE98367. ChIP-seq signals were analyzed at ATAC-seq clusters previously identified in THP-1 cells. Boxplots represent normalized tag counts for ChIP-seq (right). (C and D) Boxplot comparing normalized tag counts for each cluster using IFN-γ-primed HMDM. (E) IGV genome browser tracks show ATAC-seq regions from THP-1 cells and ChIP-seq signals from HMDM at JAKi-sensitive and -insensitive ACR. JAKi-sensitive or -insensitive ACR identified from THP-1 ATAC-seq are highlighted with colored boxes. ATAC-seq and ChIP-seq conditions are shown on the left (R, Resting macrophages; G, IFN-γ-primed macrophages). Boxes encompass the twenty-fifth to seventy-fifth percentile changes. Whiskers extend to the tenth and ninetieth percentiles. The central horizontal bar represents the median. Error bars represent means ± SD. *p* < 0.05(∗), *p* < 0.01(∗∗), *p* < 0.001(∗∗∗) and *p* < 0.0001(∗∗∗∗) by an unpaired t-test.

To explore the impact of JAK inhibition on TF binding across different ACR clusters, we analyzed ChIP-seq data (GEO: GSE43036, GEO: GSE120943 and GEO: GSE98367) from IFN-γ-primed macrophages.^41^^,^^44^ This analysis revealed distinct STAT1 and STAT3 binding patterns across the three ACR clusters, with both “JAKi-sensitive” and “JAKi-insensitive” clusters exhibiting increased binding in IFN-γ-primed macrophages compared to resting macrophages (Figure 5A). Notably, IRF1 ChIP-seq signals were predominantly enriched in the “JAKi-sensitive” ACR cluster of IFN-γ-primed macrophages (Figure 5A), corroborating the *de novo* motif analysis results (Figure 3). Analysis of normalized tag counts demonstrated that the JAKi-sensitive cluster exhibited significantly elevated binding signals for both STAT1 and IRF1 compared to the JAKi-insensitive cluster, while STAT3 binding intensities remained comparable between the clusters (Figures 5A and 5C).

We also examined the binding preferences of other TF. PU.1 preferentially bound to ACR in the “closed IFN-γ” cluster, while C/EBPβ showed strongest binding to ACR in the “JAKi-insensitive” cluster of IFN-γ-primed macrophages (Figures 5B, 5D, and S6A). Representative gene tracks further illustrated the preferential occupancy of IRF1 and STAT1 at ACR of JAKi-sensitive genes, such as *IDO1* and *GBP5* (Figures 5E and S6B). In contrast, C/EBPβ strongly bound to *cis*-regulatory regions of JAKi-insensitive genes, including *HCAR2* and *GSAP* (Figures 5E and S6C). These findings indicate that JAK inhibition selectively suppresses IRF1/STAT1-bound open chromatin regions of JAKi-sensitive genes, while JAKi-insensitive ACR preferentially interact with C/EBPβ and potentially AP-1. This differential TF binding pattern provides mechanistic insight into the selective effects of JAK inhibition on specific gene sets in IFN-γ-primed macrophages.

Among the analyzed TF, STAT3 exhibited enhanced binding following IFN-γ stimulation, despite showing minimal binding differences between JAKi-sensitive and -insensitive clusters. Based on STAT3’s established role in negatively regulating IFN responses,^45^ we investigated its potential influence on JAKi sensitivity using SD-36, a selective STAT3 degrader. Although SD-36 effectively reduced STAT3 protein levels without compromising THP-1 monocyte proliferation or STAT1 activation (Figures S6D and S6E), it had minimal effects on the expression of both JAKi-sensitive and -insensitive genes (Figure S6F). These results indicate that STAT3 does not significantly contribute to the differential expression patterns observed between JAKi-sensitive and -insensitive genes.

### Janus kinases inhibitors sensitivity distinguishes interferon-γ signature genes in rheumatoid arthritis synovial macrophages

**Figure 6:**
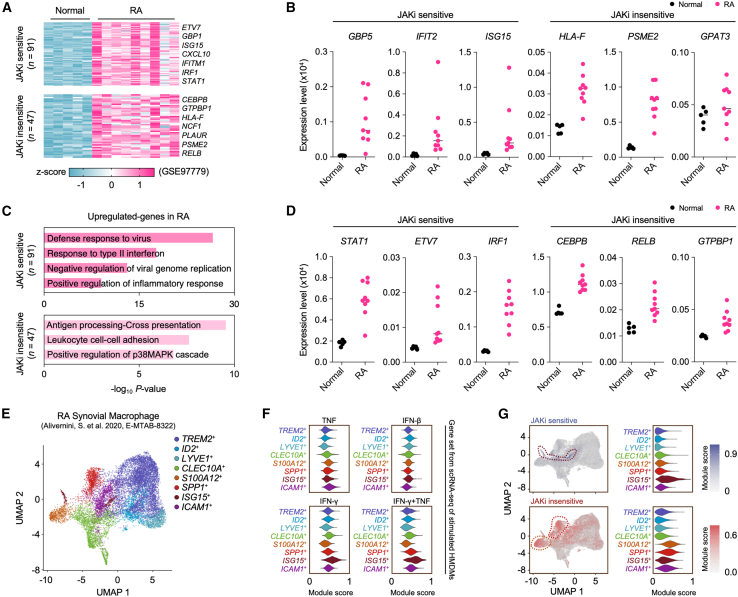
Differential regulation by distinct JAKi in the inflammatory milieu (A) Analysis of synovial tissue macrophages from patients with RA using JAKi-sensitive and -insensitive gene signatures defined in THP-1 cells. DEGs were identified using GEO2R (FDR adjusted *p* < 0.05, |Fold change| > 2). (B) Expression profiles of JAKi-responsive genes from THP-1 analysis in synovial tissue macrophages from normal controls and patients with RA (FDR adjusted *p* < 0.05, |Fold change| > 2). (C) GO analysis using JAKi-sensitive and -insensitive genes in patients with RA. Heatmap displays the significance (-log_10_*p* value) of GO term enrichment for genes in each gene set: JAKi-sensitive (*n* = 91), JAKi-insensitive (*n* = 47). GO analysis was performed using Metascape. (D) Expression profiling of JAKi-sensitive and -insensitive in synovial tissue macrophages from normal controls and patients with RA (FDR adjusted *p* < 0.05, |Fold change| > 2). (E) UMAP plot visualization of scRNA-seq data from RA synovial macrophage subpopulation (ArrayExpress: E-MTAB-8322) based on reported markers. (F) Cytokine module score analysis based on gene sets obtained from scRNA-seq of identified stimulated HMDM. Violin plots represent the distribution of cytokine module scores for the indicated cell types. (G) Module scores of JAKi-sensitive and -insensitive genes in synovial macrophage subpopulations. The color gradient in the UMAP plot indicates the strength of the module scores (left). Violin plot represents the distribution of JAKi responsiveness module scores for indicated cell types (right).

To investigate the clinical relevance of the IFN-γ signature and JAKi sensitivity, we analyzed gene expression data (GEO: GSE97779) from RA synovial macrophages.^25^ This analysis identified 91 JAKi-sensitive and 47 JAKi-insensitive genes in RA synovial macrophages (Figures 6A and S7A). GO analysis revealed that JAKi-sensitive genes were predominantly associated with “IFN- and virus-related regulation” (e.g., *GBP5*, *IFIT2*, and *ISG15*), whereas JAKi-insensitive genes were enriched for “antigen processing and cross-presentation” (e.g., *HLA-F*, *PSME2*, and *TAP2*) and “MAPK cascade” regulation (Figures 6B and 6C). Both JAKi-sensitive and JAKi-insensitive gene sets were significantly upregulated in RA compared to OA synovial macrophages (Figure S7B).^46^ This upregulation was further supported by ATAC-seq data analysis,^43^ which revealed higher chromatin accessibility for both JAKi-sensitive (*GBP5*) and JAKi-insensitive (*GPAT3*) genes in patients with RA compared to patients with OA (Figure S7C). Importantly, TF previously identified in our *de novo* motif analysis as JAKi-sensitive or JAKi-insensitive were distinctly upregulated in RA synovial macrophages (Figure 6D). These findings collectively indicate that the IFN-γ signature and differential JAKi sensitivity observed in our *in vitro* studies are reflected in the transcriptional and epigenetic landscape of RA synovial macrophages.

To further elucidate the relevance of JAKi sensitivity in RA, we analyzed scRNA-seq data from peripheral blood mononuclear cells (PBMC) (GEO: GSE159117) of patients with RA (Figures S7D and S7E).^47^ Myeloid cell populations in both PBMC and synovial fluids showed high expression of *IFNGR1*, *JAK1*, and *JAK2* (Figure S7F). Analysis of the IFN-II signaling pathway network revealed that IFN-γ, primarily secreted by NK cells, had distinct effects on CD14^+^ monocytes, CD16^+^ monocytes, and dendritic cells. (Figure S7G). Notably, CD14^+^ classical monocytes in PBMC from patients with RA exhibited the highest module score for IFN-γ signature genes (Figure S7H).

To delineate the complexity of synovial macrophages in RA, we analyzed scRNA-seq data (ArrayExpress: E-MTAB-8322), classifying macrophages into eight distinct subgroups based on previously reported gene expression patterns (Figure 6E).^3^ We then performed modular analysis using marker genes of macrophages exposed to IFN-γ, TNF, IFN-β, or combined IFN-γ+TNF (Figure S5A) to assess cytokine response profiles across these subsets. Notably, *ISG15*^+^ macrophages, previously associated with tissue infiltration, exhibited markedly increased IFN-γ and IFN-γ+TNF module scores. Concurrently, *SPP1*^+^ macrophages showed enrichment for IFN-γ+TNF module, and *S100A12*^+^ showed relative enrichment for the TNF module (Figure 6F). We next examined JAK inhibition sensitivity across these subsets. *ISG15*^+^ macrophages displayed the most pronounced JAKi-sensitive module scores, characterized by the elevated expression of JAKi-sensitive genes, such as *CXCL10* and *GBP5* (Figures 6G and S7I). In contrast, *S100A12*^+^, *SPP1*^+^, and *ICAM1*^+^ macrophages exhibited relatively high JAKi-insensitive module scores (Figure 6G). *S100A12*^+^ and *SPP1*^+^ macrophages predominantly expressed JAKi-insensitive genes (e.g., *S100A9* and *ITGB7*) and were enriched with TNF signature genes (Figures 6G and S7I). These findings highlight the heterogeneous expression patterns of JAKi-sensitive and -insensitive genes across diverse macrophage subsets in RA synovial fluid. The identification of macrophage subsets with differential responses to cytokine stimuli and JAKi sensitivity provides insight into the complex cellular landscape of the RA joint microenvironment.

### Distinct epigenetic regulatory landscapes underlie Janus kinases inhibitors sensitivity in rheumatoid arthritis synovial macrophage subsets

**Figure 7:**
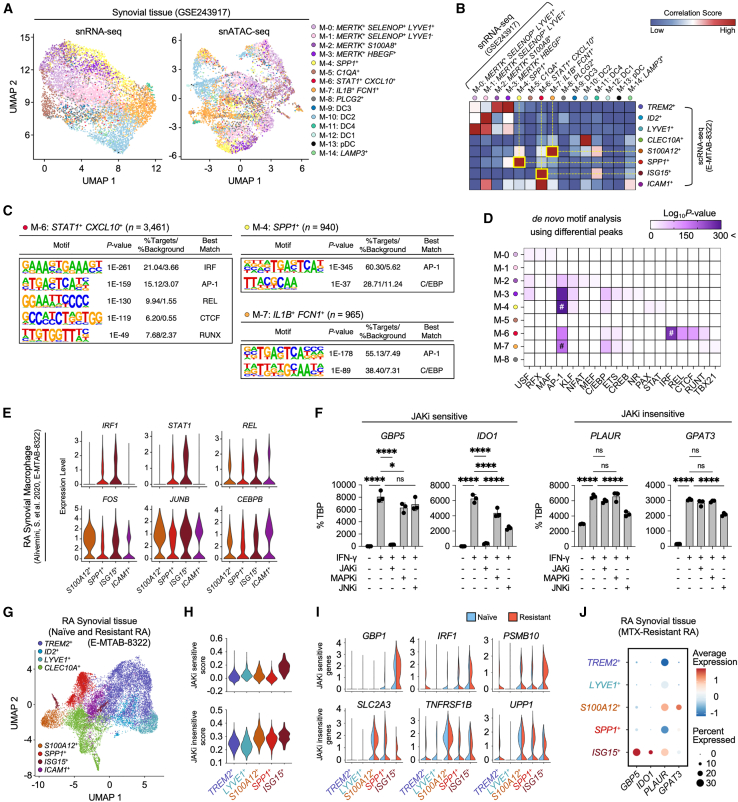
JAK inhibitor responsiveness is associated with differential chromatin accessibility in RA synovial macrophage subsets (A) UMAP plot visualization of multiome (snRNA-seq and snATAC-seq) data of a synovial macrophage subpopulation (GEO: GSE243917) from patients with arthritis (11 with RA and 1 with OA). Each cluster is based on reported cell clustering. (B) Heatmap showing significant overlap of marker genes between two published datasets (ArrayExpress: E-MTAB-8322 and GEO: GSE243917). The Jaccard score represents the percentage of pairwise overlapping genes. Cell types with the highest correlation scores are indicated by yellow boxes. (C) Most enriched TF motifs identified by *de novo* motif analysis using HOMER from marker ACR (*p*-value <0.05, log_2_FC > 0.25) in each highly correlated synovial macrophage type. (D) Heatmap of *de novo* motif analysis using cell type-specific ACR identified from synovial macrophage subpopulations. Color intensity represents the *p*-value (-log_10_). Hashtags represent motifs with the highest *p*-values in the identified cell types. (E) Expression profiles of specific genes for different synovial tissue-infiltrating macrophage subpopulations in synovial tissue. (F) Expression analysis of JAKi-sensitive and JAKi-insensitive genes in THP-1-derived macrophages under IFN-γ-primed conditions, with and without MAPK or JNK inhibitor treatment. Three independent experiments were performed (*n* = 3). (G) UMAP visualization depicting synovial tissue macrophage clusters isolated from patients with treatment-naive and JAKi-resistant RA (ArrayExpress: E-MTAB-8322). (H) Comparison of JAKi sensitivity scores across distinct synovial macrophage subpopulations from patients with treatment-naive and JAKi-resistant RA. (I) Gene expression profiles of selected targets within macrophage subpopulations exhibiting differential JAKi sensitivity. (J) Dot plot analysis showing both the mean expression levels and the percentage of cells expressing selected genes in synovial tissue obtained from patients with MTX-resistant RA. Data are presented as mean ± SD, with statistical significance determined by one-way ANOVA followed by Tukey’s multiple comparisons test (ns, not significant; ∗*p* < 0.05; ∗∗*p* < 0.01; ∗∗∗*p* < 0.001; ∗∗∗∗*p* < 0.0001).

To investigate whether diverse open chromatin landscapes in RA synovial macrophage subpopulations determine distinct patterns of JAKi sensitivity-related gene expression, we analyzed public single-nucleus (sn) multiome data (GEO: GSE243917; snRNA-seq + snATAC-seq) of synovial tissue macrophages.^48^^,^^49^ This analysis identified 15 distinct myeloid cell subpopulations in synovial tissue (Figure 7A). Using Jaccard similarity, we matched *SPP1*^+^, *ISG15*^+^, and *S100A12*^+^ macrophage subpopulations from scRNA-seq data (ArrayExpress: E-MTAB-8322) to the M-4, M-6, and M-7 subpopulations in the snMultiome data, respectively (Figure 7B). *De novo* motif analysis of differential ACR (log_2_FC > 2, *p* < 0.05) in these subpopulations unveiled distinct motif enrichment patterns. The M-6 subpopulation, corresponding to the *ISG15*^+^ subpopulation with a high JAKi-sensitive module, showed enrichment of IRF motifs (Figures 7C and 7D). In contrast, the M-4 and M-7 subpopulations, linked to *SPP1*^+^ and *S100A12*^+^ subpopulations with high JAKi-insensitive modules, exhibited enrichment of AP-1 and C/EBP motifs. Expression analysis of key TF revealed that *IRF1* and *STAT1* were highly expressed in *ISG15*^+^ and *SPP1*^+^ macrophage subpopulations. However, *REL*, *FOS*, *JUNB*, and *CEBPB* expression was not exclusive to *SPP1*^+^ and *S100A12*^+^ subpopulations (Figure 7E). These results suggest that JAKi sensitivity in RA synovial macrophage subpopulations correlates with distinct motif enrichment in their open chromatin landscapes.

Based on the association of JAKi-insensitive genes with MAPK cascade-related functions and AP-1 motif enrichment, we investigated the effects of p38 MAPK and JNK inhibitors in IFN-γ-primed macrophages. Our analysis revealed distinct inhibition patterns across gene categories. The JAKi-sensitive gene *GBP5* showed minimal response to p38 MAPK inhibition and demonstrated resistance to JNK inhibition. Notably, although classified as JAKi-sensitive, *IDO1* exhibited significant suppression in response to both p38 MAPK and JNK inhibition. Conversely, the JAKi-insensitive genes *PLAUR* and *GPAT3*, which remained largely unaffected by JAK inhibition, displayed selective and substantial suppression under JNK inhibition while maintaining resistance to p38 MAPK inhibition (Figure 7F). These findings demonstrate distinct epigenetic regulatory mechanisms associated with JAKi sensitivity in RA synovial macrophage subsets and identify JNK inhibition as a potential therapeutic strategy for targeting JAKi-insensitive genes.

To investigate potential JAKi responsiveness in treatment-resistant RA, we analyzed scRNA-seq data from synovial macrophages isolated from patients with MTX-resistant and untreated naive RA (Figure 7G). Our analysis focused on previously characterized macrophage populations: *TREM2*^+^ and *LYVE1*^+^ macrophages, which express remission-associated transcriptomic signatures, and *S100A12*^+^, *SPP1*^+^, and *ISG15*^+^ populations, which were predominantly present in MTX-resistant patients. Transcriptional profiling revealed distinct patterns of JAKi sensitivity across these populations. *ISG15*^+^ macrophages maintained elevated JAKi-sensitive scores, while *S100A12*^+^, *SPP1*^+^, and *ISG15*^+^ populations demonstrated high JAKi-insensitive scores (Figure 7H). In MTX-resistant patients, *ISG15*^+^ macrophage populations exhibited the sustained expression of JAKi-sensitive genes (*GBP1*, *IRF1*, and *PSMB10*), whereas *S100A12*^+^, *SPP1*^+^, and *ISG15*^+^ macrophage populations maintained expression of JAKi-insensitive genes (*SLC2A3*, *TNFRSF1B*, and *UPP1*) (Figure 7I). Additionally, *ISG15*^+^ macrophages showed persistent expression of JAKi-sensitive genes *GBP5* and *IDO1*, while *S100A12*^+^ macrophages specifically maintained the elevated expression of JAKi-insensitive genes *PLAUR* and *GPAT3* in MTX-resistant patients (Figure 7J). These distinctive gene expression patterns suggest that patients with MTX-resistant RA may exhibit heterogeneous responses to subsequent JAKi therapy.

## Discussion

The IFN signature is a hallmark of chronic inflammatory diseases such as RA and SLE.^16^^,^^27^^,^^50^ In inflammatory environments, IFN stimulation activates the JAK-STAT signaling pathway, which can be effectively inhibited by JAKi.^51^^,^^52^ However, the precise epigenetic mechanisms underlying the JAKi regulation of gene expression in macrophages remain unclear. Here, we investigated the epigenetic mechanisms by which JAKi regulates IFN-γ signature genes in human macrophages. Our findings provide insights into the molecular mechanisms underlying the IFN signature and JAKi effects, enhancing our understanding of JAK inhibition in diverse inflammatory conditions.

Inflammatory cytokines induce diverse responses in macrophages, leading to heterogeneous phenotypes in inflamed tissues.^9^^,^^12^ IFN-γ is particularly important in polarizing M1-like inflammatory macrophages, which are prevalent in chronic inflammatory diseases.^13^^,^^16^ Our comparative analysis demonstrated that the IFN-γ signature comprises both uniquely induced genes and genes shared with other inflammatory stimuli, such as type I IFN and TNF. These IFN-γ signature genes were predominantly expressed in patients with RA, IBD, and COVID-19.^4^^,^^53^ Single-cell transcriptome analysis revealed enrichment of IFN-γ signature genes in specific cell subpopulations linked to disease severity in both RA monocytes and synovial macrophages.^49^^,^^54^ These results implicate IFN-γ signature genes as key players in the pathogenesis of chronic inflammatory diseases.

The prolonged exposure of macrophages to environmental stimuli, including cytokines, can lead to memory-like changes known as “innate immune memory” or “trained immunity.”^55^^,^^56^^,^^57^ The IFN-γ signature is crucial in this context, associated with persistent transcriptomic and epigenomic changes that promote chronic inflammation and treatment resistance. IFN-γ priming amplifies inflammatory cytokine responses while suppressing homeostatic and anti-inflammatory gene programs.^41^^,^^44^ Our research demonstrates that JAKi-induced transcriptomic changes are shaped by epigenomic signatures in IFN-γ-primed macrophages. JAKi effectively suppresses a subset of IFN-γ-induced genes associated with IFN signaling and antiviral responses. However, certain IFN-γ-induced genes related to TLR4 signaling and the MAPK cascade remain JAKi-insensitive, despite exhibiting STAT1 and STAT3 binding at promoters or enhancers. This differential response is attributed to distinct DNA binding motifs in CRE: JAKi preferentially targets IRF-STAT motifs, while JAKi-insensitive genes contain AP-1 and C/EBP motifs. Recent studies on adjuvanted influenza vaccination have demonstrated that increased chromatin accessibility in IRF-enriched regions, relative to AP-1-enriched regions, is critical for training CD14^+^ monocytes and enhancing antiviral immunity in myeloid cells.^58^^,^^59^ A compelling finding from our study is that certain IRF motif-containing chromatin regions maintain their accessibility despite JAK inhibition. This observation reveals the intricate relationship between JAK-STAT and IRF-mediated signaling in IFN-γ-primed macrophages, where the differential enrichment of IRF motifs in JAKi-sensitive and -insensitive regions suggests a sophisticated regulatory network. The stability of IRF3 under JAK inhibition aligns with previous research demonstrating that JAKi do not directly impair IRF3 activation.^42^ We hypothesize that this sustained chromatin accessibility is maintained through complex cooperative interactions between IRF family members and other TF, notably AP-1 and C/EBPβ. Such a mechanism could serve as a critical adaptation allowing IFN-γ-primed macrophages to maintain their response capacity to subsequent inflammatory stimuli. While further investigation is needed to fully characterize these molecular interactions, this functional segregation between JAK-dependent and -independent pathways offers a potential explanation for the persistence of certain inflammatory responses during JAKi therapy. These insights into the selective regulation of IFN-γ-induced genes by JAKi through distinct epigenomic landscapes significantly advance our understanding of innate immune memory and chronic inflammation. Our findings have important implications for therapeutic strategies targeting inflammatory conditions and suggest potential mechanisms of treatment resistance.

The balance between STAT1 and STAT3 is critical in regulating inflammatory responses in macrophages, as these TF share similar DNA binding motifs and can compete for the same sites or form heterodimers and homodimers.^60^^,^^61^ Our findings reveal that while IFN-γ priming predominantly activates STAT1, it also activates STAT3. We demonstrate that the binding preferences of STAT1 and STAT3 partner TF are dictated by DNA binding motifs in distinct open chromatin regions. ChIP-seq analysis demonstrated that IRF1, a key STAT1 binding partner known to regulate TLR responses by increasing chromatin accessibility,^61^ preferentially binds to open chromatin regions of JAKi-sensitive genes along with STAT1. In contrast, C/EBPβ, a lineage-determining TF in macrophages,^62^ predominantly occupies JAKi-insensitive open chromatin regions. These distinct binding preferences likely contribute to the differential chromatin accessibility patterns observed following JAK inhibition. Although previous research has implicated STAT3 in the negative regulation of IFN responses,^45^ our experiments utilizing the STAT3-specific degrader SD-36 indicate that STAT3 may not significantly influence JAKi sensitivity within our identified gene sets. Nevertheless, the potential role of STAT3 in modulating other IFN-γ-responsive genes remains an important area for future investigation.

Importantly, we observed that IFN-γ priming can trigger autocrine loops mediated by chemokines or low levels of TNF and TNFSF members. This activation pattern results in the enrichment of specific DNA binding motifs for AP-1, C/EBP, and NF-κB within the regulatory regions of JAKi-insensitive genes, demonstrating a sophisticated network of transcriptional regulation. These observations provide critical insights into how the coordinated actions of TF and cytokine signaling pathways shape the epigenomic landscape of IFN-γ-primed macrophages. In addressing potential therapeutic strategies for JAKi resistance, we evaluated the effects of JNK inhibition in IFN-γ-primed macrophages. Our findings demonstrate that JNK inhibition effectively suppresses JAKi-insensitive genes, including *PLAUR* and *GPAT3*. This discovery suggests that JNK inhibition could serve as a complementary therapeutic approach to enhance the efficacy of JAK inhibition in inflammatory conditions by targeting otherwise resistant gene networks.

Single-cell analysis of RA synovial macrophages revealed distinct patterns of JAKi sensitivity across subpopulations. JAKi-sensitive genes were preferentially expressed in the *ISG15*^+^ subpopulation, while JAKi-insensitive genes showed high expression in *SPP1*^+^ and *S100A12*^+^ subpopulations. Single-cell ATAC-seq data analysis corroborated these findings, demonstrating that open chromatin regions in the *ISG15*^+^ subpopulation were enriched for IRF1 and STAT1 motifs and gene expression. Conversely, *SPP1*^+^ and *S100A12*^+^ subpopulations exhibited enrichment of AP-1 and C/EBP motifs. Intriguingly, the *ISG15*^+^ macrophage subpopulation also expressed certain JAKi-insensitive genes, including those associated with the IFN-γ+TNF module and AP-1 motifs. This observation suggests that JAK inhibition might not completely suppress the *ISG15*^+^ subpopulation but rather preferentially target JAKi-sensitive genes. Furthermore, JAKi may not effectively inhibit the inflammatory *SPP1*^+^ and *S100A12*^+^ subpopulations, indicating that alternative strategies, such as TNF or JNK inhibition, might be necessary for comprehensive targeting of all inflammatory macrophage subsets in RA. These findings highlight the complex heterogeneity of synovial macrophages in RA and the differential effects of JAK inhibition across subpopulations.

JAKi have emerged as a crucial therapeutic option for patients with RA who demonstrate resistance to MTX treatment.^52^ Our single-cell transcriptomic analysis of synovial macrophages has unveiled significant molecular heterogeneity in IFN-γ responses among patients with RA, characterized by distinct expression profiles of JAKi-sensitive and -insensitive genes. The identification of JAKi-sensitive genes, including *GBP1* and *PSMB10*, presents promising therapeutic targets for JAKi therapy in patients with MTX-resistant RA. Conversely, the expression of JAKi-insensitive genes, such as *SLC2A3* and *UPP1*, may contribute to treatment resistance and influence the safety profile of JAKi therapy. These observations suggest that drug resistance in RA is fundamentally linked to the differential regulation of IFN-γ signature genes and their varied responsiveness to JAK inhibition.^63^ The complexity of these regulatory mechanisms across different clinical contexts of human diseases warrants further investigation to fully elucidate the mechanistic basis of the differential JAKi regulation of IFN-γ signature genes.^64^^,^^65^^,^^66^ Such understanding could lead to more effective therapeutic strategies for patients with treatment-resistant inflammatory conditions.

In this study, we uncover the epigenetic mechanisms underlying the JAKi regulation of IFN-γ signature genes in human macrophages. Our findings reveal a complex interplay between TF, chromatin accessibility, and JAK-STAT signaling that determines the sensitivity of specific gene sets to JAK inhibition. We demonstrate that JAKi-sensitive and JAKi-insensitive genes are associated with distinct TF motifs and chromatin landscapes. This differential regulation has important implications for the efficacy and potential limitations of JAKi therapy in chronic inflammatory diseases. The identification of JAKi-insensitive gene modules and their associated regulatory mechanisms opens avenues for combination therapies that could enhance the effectiveness of JAK inhibition. These insights not only deepen our understanding of the IFN signature’s epigenetic regulation but also illuminate potential avenues for more targeted and effective JAKi therapies across a spectrum of inflammatory conditions.

### Limitations of the study

Our study has several limitations. First, our experiments utilized only a pan-JAKi (tofacitinib) to examine the effects of JAK inhibition on IFN-γ signature gene regulation. Future studies should investigate JAK1-selective inhibitors, such as upadacitinib and filgotinib, to provide a more comprehensive understanding of how different JAKi modulate IFN-γ signature genes in various clinical contexts, including patients with refractory RA. Second, our study focused primarily on IFN-γ signaling, but we cannot exclude the potential combined effects of other cytokines present in disease environments. For instance, high levels of IL-6 in inflamed tissues might activate STAT3 in IFN-γ-primed macrophages. Further exploration is necessary to understand the interplay between various cytokines, such as interleukin-6 (IL-6) and IFN-γ, on macrophages in the context of epigenomic mechanisms. Lastly, while our single-cell analysis revealed differential JAKi sensitivity across macrophage subpopulations in RA synovial tissue, these findings do not provide a complete picture of drug responsiveness for individual patients. Additional research is crucial to fully elucidate the mechanisms by which JAKi differentially regulate IFN-γ signature genes across diverse clinical contexts of human diseases.

## Resource availability

### Lead contact

Further information and requests for resources and reagents should be directed to and will be fulfilled by the lead contact, Kyuho Kang (kangk@cbnu.ac.kr).

### Materials availability

This study did not generate new unique reagents.

### Data and code availability

The datasets generated by the authors will be deposited in the Gene Expression Omnibus (GEO) database under the accession number GEO: GSE244130. The analysis was performed using standard bioinformatic tools with parameters described in the STAR Methods section. No custom algorithms were developed for this study.

## Acknowledgments

We thank Dr. Lionel Ivashkiv for helpful discussions and review of the article. This work was supported by the 10.13039/501100001321National Research Foundation (NRF) of Korea grant funded by the Korea government (MSIT) (NRF-2020R1C1C1013939 and RS-2024-00340411), 10.13039/501100002461Chungbuk National University BK21 program (2023), and grants from the 10.13039/100000002NIH (R01AR083374 and R01AR073156).

## Author contributions

Conceptualization, G.K., Kyuho K., and K.-H.P.-M.; methodology, G.K.; Investigation, G.K. and Y.P.; formal analysis, G.K., Y.P., and Keunsoo K.; writing – original draft, G.K.; writing – review and editing, Kyuho K., K.-H.P.-M., and all authors; supervision, Kyuho K. and K.-H.P.-M.

## Declaration of interests

The authors declare no competing interests.

## STAR★Methods

### Key resources table

**Table:** 

REAGENT or RESOURCE	SOURCE	IDENTIFIER
**Antibodies**		

Alexa Fluor® 647 Mouse anti-Total Stat1	BD biosciences	Cat# 558560; RRID: AB_647143
PE anti-STAT1 Phospho (Tyr701)	BioLegend	Cat# 666404; RRID: AB_2734526
PE anti-human STAT3	BioLegend	Cat# 371804; RRID: AB_2629734
Brilliant Violet 421 anti-STAT3 Phosph (Tyr705)	BioLegend	Cat# 651010; RRID: AB_2572088

**Chemicals, peptides, and recombinant proteins**		

RPMI-1640 medium (1X)	Gibco™	Cat# 11875-119
Fetal Bovine Serum, certified, United States	Gibco™	Cat# 16000-044
Penicillin/streptomycin, Liquid, 5000ug/ml	Gibco™	Cat# 15070-063
Phorbol 12-myristate 13-acetate	Sigma-Aldrich	Cat# P1585
Recombinant Interferon-γ, human (hIFN-γ)	Roche	Cat# 11040596001
Deoxyribonuclease I	Worthington	Cat# LS002004
Fixation buffer	BioLegend	Cat# 420801
True-Phos™ Perm	BioLegend	Cat# 425401
Cell staining buffer	BioLegend	Cat# 420201
Dulbecco’s phosphate buffered saline	BIOWEST	Cat# L0615-500
Trypan Blue Solution, 0.4%	Gibco™	Cat# 15250061
TrypLE™ Express (1X), no Phenol Red	Gibco™	Cat# 12604013
Tofacitinib citrate, JAK inhibitor	Sigma-Aldrich	Cat# PZ0017-5MG
Adezmapimod, p38 MAPK inhibitor	Medchemexpress	Cat# HY-10256
SP600125, JNK inhibitor	Medchemexpress	Cat# HY-12041
SD-36 (PROTAC STAT3 Degrader)	MedChemExpress	Cat# HY-129602
Digitonin	Promega	Cat# G9441
NP40	Roche	Cat# 11332473001
Tween-20	Roche	Cat# 11332465001
1M Tris-HCl pH 7.5	Invitrogen™	Cat# 15567-027
5M NaCl	Invitrogen™	Cat# AM9759
1M MgCl2	Invitrogen™	Cat# AM9530G
NEBNext® High-Fidelity 2X PCR Master Mix	NEB	Cat# M0541L
Nuclease-Free Water (10 × 50 ml)	QIAGEN	Cat# 981103
2-Mercaptoethanol	Sigma-Aldrich	Cat# M3148-25ML
Ethanol absolute for analysis EMSURE	Supelco	Cat# 1.00983.1000
TD Tagment DNA Buffer	Illumina	Cat# 15027866
TDE1 Tagment DNA Enzyme	Illumina	Cat# 15027865

**Critical commercial assays**		

Ribospin™ II	GeneAll Biotechnology	Cat# 314-150
RevertAid First Strand cDNA Synthesis kit	ThermoFisher™	Cat# K1622
MinElute Reaction Cleanup Kit	QIAGEN	Cat# 28206
TOPreal™ qPCR 2X PreMIX	Enzynomics	Cat# RT500M

**Deposited data**		

Microarray data: Synovial macrophages (RA)	Kang et al., 2017^25^	GEO: GSE97779
RNA-seq: IFN-γ-primed HMDM	Kang et al., 2017^25^	GEO: GSE98368
RNA-seq: IFN-α-stimul HMDM	Park et al., 2017^6^	GEO: GSE100382
RNA-seq: TNF-stimul HMDM	Park et al., 2017^6^	GEO: GSE100382
RNA-seq: THP-1 monocyte-derived macrophages	This paper	GEO: GSE244129
ChIP-seq: STAT1 and IRF1	Qiao et al., 2013^41^	GEO: GSE43036
ChIP-seq: PU.1 and C/EBPβ	Kang et al., 2017^25^	GEO: GSE98367
ChIP-seq: STAT3	Kang et al., 2019^44^	GEO: GSE120943
ChIP-seq: NF-κB	Park et al., 2017^6^	GEO: GSE100381
ATAC-seq: IFN-γ-primed HMDM	Kang et al., 2017^25^	GEO: GSE98365
ATAC-seq: Monocytes from OA and RA patients	Zong, Dandan et al. 2021^43^	NGDC: CRA002749
ATAC-seq: THP-1 monocyte-derived macrophages	This paper	GEO: GSE244128
Single-cell RNA-seq: Blood-derived macrophages	Zhang et al., 2021^4^	GEO: GSE168710
Single-cell RNA-seq: PBMC (RA)	Zhang et al., 2022^47^	GEO: GSE159117
Single-cell RNA-seq: Synovial tissue (RA)	Alivernini et al., 2020^3^	ArrayExpress: E-MTAB-8322
snMultiome: Synovial tissue (RA)	Gupta, Anika et al. 2023^48^	GEO: GSE243917
AMP Rheumatoid Arthritis Phase 1	Zhang, Fan et al. 2019^46^	ImmPort: SDY998

**Experimental models: Cell lines**		

THP-1	Korean Cell Line Bank	Cat# KCLB 40202

**Software and algorithms**		

R package: DESeq2 v1.28.1	Love et al., 2014^67^	https://bioconductor.org/packages/release/bioc/html/DESeq2.html
R package: edgeR v3.30.3	McCarthy et al., 2012^68^	http://bioconductor.org/packages/release/bioc/html/edgeR.html
R package: Seurat v4.1.0	Hao et al., 2021^69^	https://satijalab.org/seurat/index.html
R package: ggplot2 v3.3.6	Wickham, 2016^70^	https://ggplot2.tidyverse.org/
R package: ggfortify v0.4.14	Yuan et al., 2016^71^	https://cran.r-project.org/web/packages/ggfortify/index.html
R package: ATACseqQC v1.12.5	Ou et al., 2018^72^	https://bioconductor.org/packages/release/bioc/html/ATACseqQC.html
R v4.0.3	R Core Team, 2020^73^	https://www.r-project.org/
fasterq-dump v2.10.8	SRA Toolkit	https://github.com/ncbi/sra-tools
Trim Galore v0.6.6	Trim Galore	https://github.com/FelixKrueger/TrimGalore
Bowtie2 v2.3.5.1	Langmead and Salzberg, 2012^74^	http://bowtie-bio.sourceforge.net/bowtie2/index.shtml
STAR aligner v2.7.3a	Dobin et al., 2013^75^	https://github.com/alexdobin/STAR
RSeQC v4.0.0	Wang et al., 2012^76^	http://rseqc.sourceforge.net
HOMER v4.11.1	Heinz et al., 2010^40^	http://homer.ucsd.edu/homer/index.html
Gene Set Enrichment Analysis (GSEA) v4.1.0	Mootha et al., 2003; Subramanian et al., 2005^77^^,^^78^	https://www.gsea-msigdb.org/gsea/index.jsp
GraphPad Prism v10.2.3	GraphPad Software, Inc	https://www.graphpad.com/
deepTools v3.5.0	Ramirez et al., 2016^79^	https://deeptools.readthedocs.io/en/develop/
Circos v0.69-8	Krzywinski et al., 2009^80^	http://circos.ca/
GEO2R	Barrett et al., 2013^81^	https://www.ncbi.nlm.nih.gov/geo/geo2r/
Metascape	Zhou et al., 2019^82^	https://metascape.org/gp/index.html#/main/step1
CytExpert v2.4	Beckman Coulter	https://www.beckmancoulter.com/
Integrative Genomics Viewer (IGV) v2.9.2	Robinson et al., 2011^83^	https://software.broadinstitute.org/software/igv/
Morpheus	Morpheus	https://software.broadinstitute.org/morpheus
Venny v2.1.0	Oliveros, 2007–2015^84^	https://bioinfogp.cnb.csic.es/tools/venny/index.html

### Experimental model and subject details

#### Cell culture

Human monocytic THP-1 cells (Korean Cell Line Bank) were cultured in RPMI-1640 medium (Gibco) supplemented with 10% fetal bovine serum (FBS) (Gibco) and 1% penicillin/streptomycin (Gibco). Cells were maintained in a humidified incubator at 37°C with 5% CO2. To differentiate THP-1 monocytes into macrophages, cells were treated with 100 nM phorbol 12-myristate 13-acetate (PMA) (Sigma-Aldrich) for 24 h. For the generation of IFN-γ-primed macrophages, THP-1 cells were co-treated with PMA and 100 U/ml human IFN-γ (Roche) for 24 h. Following priming, the macrophages were treated with tofacitinib (JAK inhibitor) (Sigma-Aldrich) at concentrations up to 1 μM for 6 h. Cells were then harvested for RNA-seq and ATAC-seq analyses. In a separate experiment, THP-1 monocytes were treated with adezmapimod (p38 MAPK inhibitor) (MedChemExpress) and SP600125 (JNK inhibitor) (MedChemExpress) at a concentration of 10 μM, in combination with PMA and IFN-γ, for 24 h. For SD-36 treatment experiments, cells were pretreated with 1 μM SD-36 (MedChemExpress) for 24 h prior to PMA stimulation, with or without IFN-γ.

### Method details

#### RNA extraction, cDNA synthesis, and real-time PCR

Total RNA was extracted from THP-1 monocyte-derived macrophages using the RibospinTM II kit (GeneAll Biotechnology), and 500 ng of total RNA was reverse transcribed using the RevertAid First Strand cDNA Synthesis kit (ThermoFisher). Real-time PCR was performed using the TOPreal™ qPCR 2X PreMIX (Enzynomics) and analyzed with Rotor-Gene Q software version 2.3.1 (QIAGEN). Primer sequences are listed in Table S1. RNA samples were stored at −80°C for subsequent RNA sequencing analysis.

#### Omni-ATAC-seq library preparation

The Omni-ATAC-seq library was prepared as previously described.^85^^,^^86^ THP-1 monocyte-derived macrophages (50,000 cells) were pretreated with 200 U/ml deoxyribonuclease I (Worthington) at 37°C for 30 min to remove free-floating and dead cell DNA. Cells were washed with Dulbecco’s phosphate-buffered saline (BIOWEST) and harvested using TrypLE™ (Gibco™). Harvested cells were centrifuged at 500 × g for 5 min at 4°C, washed with cold ATAC-seq resuspension buffer (RSB; 10 mM Tris-HCl pH 7.5, 10 mM NaCl, and 3 mM MgCl_2_), and centrifuged again under the same conditions. The supernatant was carefully removed, and cell pellets were resuspended in 50 μl of ATAC-seq RSB containing 0.1% NP-40 (Roche), 0.1% Tween-20 (Roche), and 0.01% digitonin (Promega). The lysis reaction was incubated on ice for 3 min followed by the addition of 1 ml ATAC-seq RSB with 0.1% Tween-20. Nuclei were centrifuged for 10 min at 500 × g at 4°C, and the supernatant was removed. Nuclei were resuspended in 50 μl of transposition mix (25 μl TD Tagment DNA Buffer (Illumina), 2.5 μl TDE1 Tagment DNA Enzyme (Illumina), 16.5 μl DPBS, 0.5 μl 1% digitonin, 0.5 μl 10% Tween-20, and 5 μl nuclease-free water) and incubated at 37°C for 30 min with shaking at 1,000 rpm. Transposed DNA was cleaned using the MinElute Reaction Cleanup Kit (QIAGEN) and pre-amplified for 5 cycles using NEBNext® High-Fidelity 2X PCR Master Mix (NEB) with Illumina/Nextera adapter primers (Table S2). The amplification profile was evaluated to determine additional cycles needed, following Buenrostro et al.^86^ The final library was purified using AMPure XP beads (Beckman) and stored at −80°C for ATAC-seq analysis.

#### Flow cytometry

THP-1 cells were fixed in pre-warmed (37°C) fixation buffer (BioLegend) at a concentration of 3×10^5^ cells for 15 minutes at 37°C. Following centrifugation at 350 × g for 5 minutes at room temperature, cells were resuspended in cell staining buffer (BioLegend). The cells were then permeabilized using pre-chilled (−20°C) True-Phos™ Perm Buffer (BioLegend) for 60 minutes at −20°C. After centrifugation at 1,000 × g for 5 minutes at room temperature, cells were washed twice with cell staining buffer. For protein detection, cells were stained separately with antibodies against either STAT1 (total STAT1: Alexa Fluor® 647 Mouse anti-Total Stat1, BD Biosciences; phospho-STAT1: PE anti-STAT1 Phospho (Tyr701), BioLegend) or STAT3 (total STAT3: PE anti-human STAT3, BioLegend; phospho-STAT3: Brilliant Violet 421 anti-STAT3 Phospho (Tyr705), BioLegend) for 30 minutes at room temperature protected from light. Following two washes with cell staining buffer, cells were analyzed using a CytoFLEX flow cytometer (Beckman Coulter). Data analysis was performed using CytExpert software.

#### Genome alignment and annotations

All human data were aligned and annotated against the *hg38* reference genome. Reads were aligned to the *hg38* genome using the hg38 genome sequence (*hg38*.fa, available at https://hgdownload.soe.ucsc.edu/goldenPath/*hg38*/bigZips/*hg38*.fa.gz) and gene annotation (*hg38*.gtf, obtained from the UCSC Table Browser) files provided by the University of California, Santa Cruz (UCSC). To annotate aligned reads, we used the “*hg38*.ncbiRefSeq.gtf” file (available at https://hgdownload.soe.ucsc.edu/goldenPath/*hg38*/bigZips/genes/*hg38*.ncbiRefSeq.gtf.gz) provided by UCSC. As a cross-validation, the annotation analysis was further verified using the default hg38 reference genome in the HOMER software.

#### Creating tag directories for bulk sequencing data

The raw sequencing data were quality filtered using Trim Galore (version 0.6.6). Preprocessed reads were mapped to the human *hg38* genome. RNA-seq data were mapped to the *hg38* genome using the STAR aligner (version 2.7.3a) with default parameters.^75^ ChIP-seq data were mapped to the *hg38* genome using Bowtie2 (version 2.3.5.1) with default parameters.^74^ ATAC-seq data were mapped to the *hg38* genome using Bowtie2 with the parameters “*–very-sensitive –no-discordant -X 2000*” (available at https://github.com/harvardinformatics/ATAC-seq, last updated: 2019). The aligned reads were converted into tag directories using the “*makeTagDirector”* command in the HOMER software (version 4.11.1) for further analysis.^40^

#### Bulk RNA-seq analysis

The RNA-seq data used in this study were obtained from the Gene Expression Omnibus (GEO) under accession numbers GEO: GSE98368, GEO: GSE100382, and from our own experiments (GEO: GSE244130).^6^^,^^25^ Mapped reads were quantified using the “*analyzeRepeats*” script of the HOMER software (version 4.11.1).^40^ Transcripts per million (TPM) values were generated using the parameters “*-count exons -condenseGenes -tpm”*, while raw read counts were obtained using “*-count exons -condenseGenes -noadj*”. DEG were identified using raw sequencing read counts by edgeR (version 3.30.3) analysis via the “*getDifferentialExpression*” HOMER command, with an adjusted *p*-value (*P*-adj) < 0.05 and |Fold change| > 2.^68^ Genes with TPM values less than 4 were removed from the list of DEG. GO analysis was performed using Metascape.^82^ Genes showing positive correlations between RNA-seq datasets were identified using Venny (version 2.1.0).^84^

For the RNA-seq data generated from our experiments (GEO: GSE244130), we performed a comprehensive analysis to identify biologically relevant clusters of DEG. First, clusters that were not significant between replicates were excluded. We then focused on three distinct clusters that were clearly separated. To validate the biological relevance of these clusters, we cross-compared our transcriptome data from THP-1 cells with publicly available data (GEO: GSE98368) from primary human macrophages.^25^ Genes that showed positive correlations with the DEG identified in datasets of resting and IFN-γ-primed macrophages were selected for subsequent analyses.

#### Bulk ChIP-seq and ATAC-seq analysis

The ChIP-seq and ATAC-seq data used in this study consisted of published data obtained from GEO under accession numbers GEO: GSE43036, GEO: GSE120943, GEO: GSE98365, GEO: GSE100381, and the data generated from our own experiments.^6^^,^^25^^,^^41^^,^^44^ For ChIP-seq and ATAC-seq analysis, preprocessed reads were aligned to reference the human genome using bowtie2 with parameters. The quality check of ATAC-seq was performed with ATACseqQC (v1.12.5).^72^ We used the “*makeTagDirectory*” followed by “*findPeaks*” command from HOMER to identify peaks of ChIP-seq enrichment over the background. A false discovery rate (FDR) threshold of 0.001 was used for all data sets. ChIP-seq and ATAC-seq peaks were used with 2-fold changes compared to controls. The bedgraph file was created using the “*makeUCSCfile*” HOMER command, with the “*-fragLength given -o auto -fsize 5e7 -res 1*” parameter. The bigwig file was created using the “*bamCoverage*” deepTools (v3.5.0) command, with the “*--binSize 10 --normalizeUsing RPGC --effectiveGenomeSize 2913022398*” parameter.^79^ ATAC-seq of THP-1 monocyte-derived IFN-γ-primed macrophages identified significant clusters using overlapping peaks for each condition. ACR regulated by JAKi in IFN-γ-primed macrophages were excluded when identifying “JAKi insensitive” clusters.

Detailed analyzes were performed to identify significant ACR clusters on ATAC-seq data generated from the experiment (GEO: GSE244130). To identify significantly regulated differential ACR, an overlap analysis was performed by comparing all conditions. “JAKi-sensitive” ACR clusters were identified by overlapping all upregulated ACR in G (ctrl). The “JAKi-insensitive” cluster was upregulated in G (ctrl and JAKi) compared to R (ctrl and JAKi) and identified not-significant ACR between G(ctrl) and G(JAKi). ACR for the “closed by IFN-γ” cluster were identified with the opposite approach for the “JAKi-insensitive” cluster. ATAC-seq data from THP-1 cells generated in this study were compared with publicly available data (GEO: GSE98365) from primary human macrophages.^25^ This comparison identified 2,774 regions positively correlated with primary human macrophages, which were used in subsequent analyses. The identified differential ACR were used for motif enrichment analysis.

#### Principal component analysis (PCA)

To perform PCA, normalized tag count values were obtained for each condition. After calculating the average value of the normalized number of tags for each condition, the top 10,000 genes (for RNA-seq) and ACR (for ATAC-seq) were selected by sorting them in descending order according to the size of the values. Visualization of the PCA plot was performed using the average normalized tag count values of the top 10,000 genes and ACR. The analysis was conducted using the R package.

#### Bulk ATAC-seq analysis of monocytes from OA and RA patients

The ATAC-seq data for monocytes from OA and RA patients was obtained from the National Genomics Data Center (NGDC) using the accession link (https://ngdc.cncb.ac.cn/gsa/browse/CRA002749) with the identifier NGDC: CRA002749.^43^ For ATAC-seq analysis, preprocessed reads were aligned to the human genome using bowtie2 or an equivalent tool with appropriate parameters. The bigwig file was created using the “*bamCoverage*” command from deepTools (v3.5.0), with the parameters *“--binSize 10 --normalizeUsing RPGC --effectiveGenomeSize 2913022398*”. For the purpose of a consolidated visual representation, bigwig files pertaining to OA and RA patient monocytes were amalgamated via the “*bigwigAverage*” command. Individual patient data, when portrayed in the Integrated Genome Viewer (IGV), was achieved through an overlay of bigwig files, offering a comprehensive view of the data landscape. ACR (*n* = 1,871) increased in RA compared to OA were identified by edgeR (FDR adjusted *p* < 0.05, Fold change >2). The identified differential ACR were used for motif enrichment analysis.

#### Single-cell RNA-seq analysis

The pre-processed gene-cell matrices of the single-cell RNA-seq data were downloaded from the GEO or EMBL’s European Bioinformatics Institute (EMBL-EBI) database (accession number GEO: GSE168710, GEO: GSE159117 and ArrayExpress: E-MTAB-8322).^3^^,^^4^^,^^47^ These matrices were subsequently loaded into Seurat (v4.1.0) R package for quality control, filtering, and downstream analyses.^69^ Cells with a mitochondrial gene percentage greater than 10%, or fewer than 200 genes or more than 6,000 genes, were filtered out. Some samples were cut off more strongly according to the reference paper. The gene expression data was normalized using a log normalization method, and PCA was performed to reduce the dimensionality of the data. The significant principal components were selected based on the “elbow plot” method, and clustering was performed using the shared nearest neighbor (SNN) algorithm. DEG between different clusters were identified using the Wilcoxon rank-sum test. Cell type identification is based on known cell type markers and was further confirmed through reference articles. The UMAP plot was generated to visualize the cell clusters and the DEG. Module scores were computed using Seurat to estimate the activity levels of given gene sets across individual cells.

#### Microarray data analysis

The microarray data used in this study used public data obtained from GEO under accession number GEO: GSE97779.^25^ Microarray data analysis was performed using the Gene Expression Omnibus 2R (GEO2R) online tool. Differential expression analysis was performed between control macrophages and RA synovial macrophages using GEO2R. DEG with FDR <0.05 and fold change >2 were considered significant.

#### snMultiome data analysis

Publicly available myeloid snRNA-seq and snATAC-seq datasets were utilized.^48^^,^^49^^,^^54^ The preprocessed .h5ad files were converted to the .h5seurat format for analysis within the R environment. These converted files were then uploaded to R using “*LoadH5Seurat*” for subsequent analysis. The labeling information present in the preprocessed data was employed to distinguish myeloid cell clusters. Using the marker genes identified within myeloid cell subpopulations, we conducted correlation analysis with other published scRNA-seq data (ArrayExpress: E-MTAB-8322) from RA synovial tissue.^3^ Correlation analysis was performed using the Jaccard Similarity Index. The markers of identified ACR within myeloid cell subpopulations were utilized for *de novo* motif analysis.

#### Motif enrichment

HOMER’s motif analysis (*findMotifsGenome*.*pl*), including known motifs and *de novo* motifs, was used to identify motifs enriched in differentially regulated ACR. To investigate the distribution of TF binding motifs in the JAKi-sensitive and -insensitive clusters, motif frequency analysis was performed. The genomic coordinates of the peaks in the JAKi-sensitive and -insensitive clusters were obtained from the ATAC-seq data. Motif frequency was then calculated using the “*annotatePeaks*.*pl”* function from the HOMER software. The motif frequency was calculated in 5-bp bins within a 600-bp window centered on the peak summit.

#### Data visualization

GraphPad Prism software was used for data visualization. The IGV (v2.9.2) and deepTools (v3.5.0) were used to visualize ChIP-seq and ATAC-seq data.^83^ RNA-seq data were visualized with heatmap using Morpheus (http://software.broadinstitute.org/morpheus).

### Quantification and statistical analysis

Statistical analyses were performed using GraphPad Prism 10. Data are presented as mean ± SD. One-way ANOVA followed by Tukey’s multiple comparisons test was used for group comparisons. Significance levels are indicated as ns (not significant), ∗*p* < 0.05, ∗∗*p* < 0.01, ∗∗∗*p* < 0.001 and ∗∗∗∗*p* < 0.0001. In box plots, bounds represent 25th-75th percentiles, center line the median, and whiskers 10th-90th percentiles. Statistical details for RNA-seq, ATAC-seq, ChIP-seq, and single-cell analyses are described in the method details section. Statistical details for specific experiments are provided in the corresponding figure legends.
