# Supplementary material for: Epigenomic landscapes define differential Janus kinases inhibitor sensitivity in IFN-γ-primed human macrophages
Source: iScience. 2025 Apr 22;28(5):112502. doi: 10.1016/j.isci.2025.112502 (PMC12146619; doi:10.1016/j.isci.2025.112502)
Supplement: Document S1. Figures S1–S9 and Tables S1 and S2 [file mmc1.pdf]

iScience, Volume *28*

Supplemental information

Epigenomic landscapes define differential Janus kinases inhibitor sensitivity in IFN-γ-primed human macrophages

Geunho Kwon, Yebin Park, Keunsoo Kang, Kyung-Hyun Park-Min, and Kyuho Kang

Figure S1

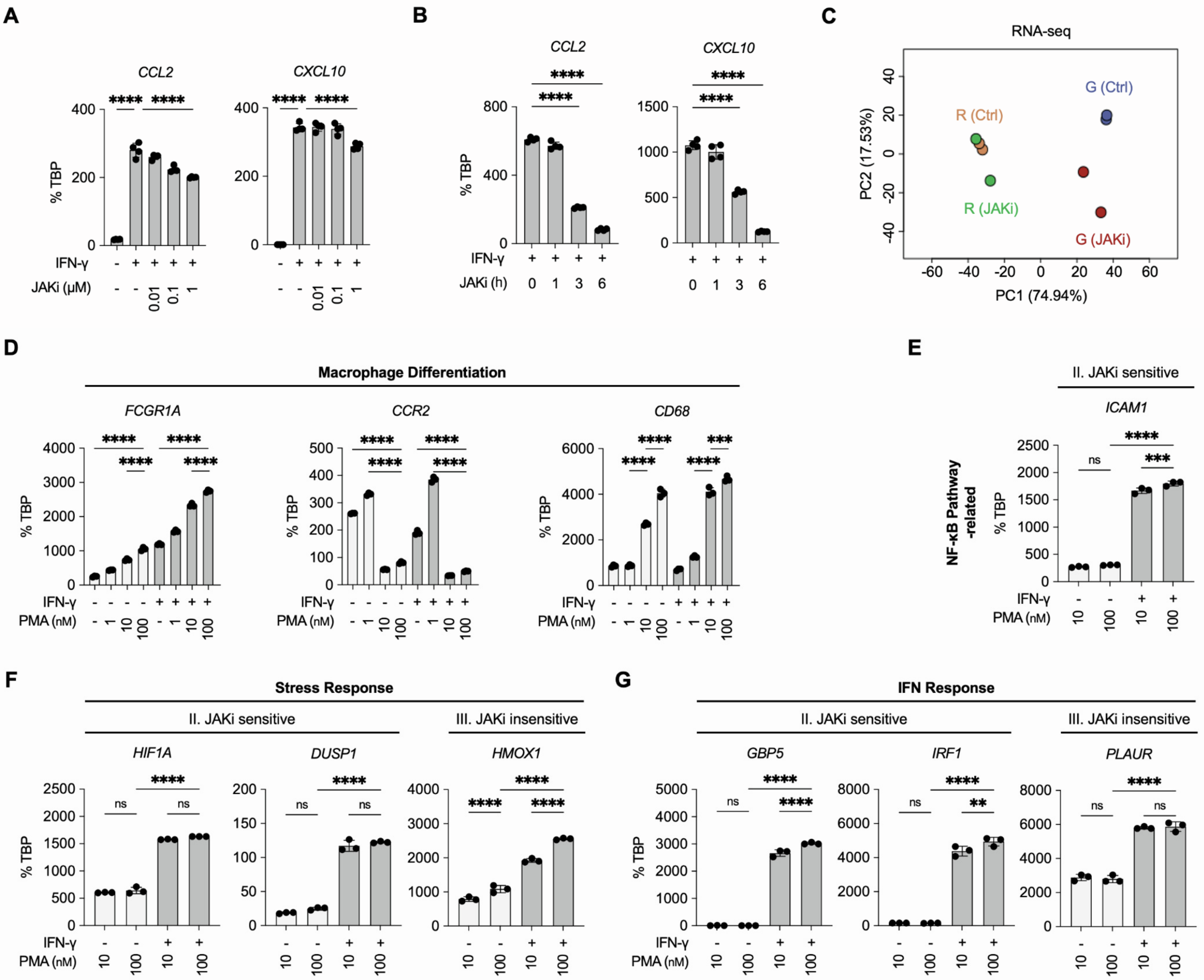

A
CCL2
CXCL10
% TBP
IFN-γ
JAKi (μM)
B
CCL2
CXCL10
% TBP
IFN-γ
JAKi (h)
C
RNA-seq
R (Ctrl)
G (Ctrl)
R (JAKi)
G (JAKi)
PC1 (74.94%)
PC2 (17.53%)
D
Macrophage Differentiation
FCGR1A
CCR2
CD68
% TBP
IFN-γ
PMA (nM)
E
II. JAKi sensitive
ICAM1
NF-κB Pathway -related
% TBP
IFN-γ
PMA (nM)
F
Stress Response
II. JAKi sensitive
III. JAKi insensitive
HIF1A
DUSP1
HMOX1
% TBP
IFN-γ
PMA (nM)
G
IFN Response
II. JAKi sensitive
III. JAKi insensitive
GBP5
IRF1
PLAUR
% TBP
IFN-γ
PMA (nM)

Figure S1. JAK inhibition modulates IFN-γ signaling and gene expression in THP-1-derived macrophages, **related to Figure 1. (A and B)** RT-qPCR analysis of target mRNA levels normalized to TBP mRNA in THP-1-derived macrophages under the indicated conditions. IFN-γ-primed macrophages were differentiated with PMA (100 nM) and IFN-γ (100 U/ml) for 24 h and treated with (A) JAKi (tofacitinib) at concentrations up to 1 μM for 1 h or (B) 1 μM JAKi for up to 6 h. **(C)** PCA results of RNA-seq samples from THP-1-derived macrophages. Total DEG (*n* = 1,709) identified by edgeR (FDR-adjusted $P < 0.05$, |Fold change| > 2) for all pairwise comparisons were used. Each point represents an RNA-seq sample. **(D)** RT-qPCR analysis of macrophage differentiation marker genes (*FCGR1A*, *CCR2*, and *CD68*) in THP-1 cells treated with indicated concentrations of PMA (0, 10, or 100 nM) with or without IFN-γ for 24 h. **(E)** Expression analysis of *ICAM1*, a JAKi-sensitive gene, in THP-1 cells treated with PMA and/or IFN-γ under the indicated conditions. **(F)** RT-qPCR analysis of stress response genes in THP-1 cells. JAKi-sensitive genes (*HIF1A* and *DUSP1*) and JAKi-insensitive gene (*HMOX1*) were examined following PMA and IFN-γ treatment. **(G)** Expression analysis of IFN response genes. JAKi-sensitive genes (*GBP5* and *IRF1*) and JAKi-insensitive gene (*PLAUR*) were measured under the indicated conditions. **(D-G)** Three independent experiments were performed (*n* = 3). Data are presented as mean ± SD, with statistical significance determined by one-way ANOVA followed by Tukey's multiple comparisons test (ns, not significant; *$P < 0.05$; **$P < 0.01$; ***$P < 0.001$; ****$P < 0.0001$).

Figure S2

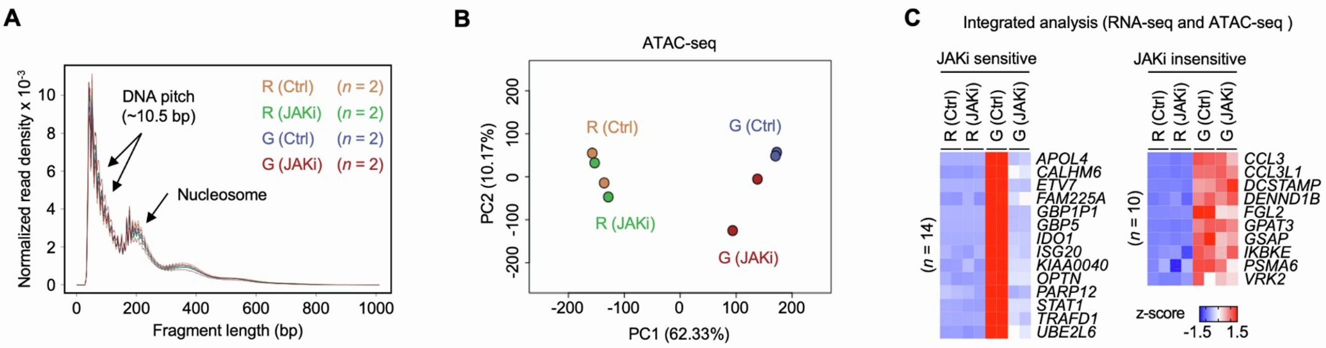
A
Normalized read density x 10⁻³
Fragment length (bp)
DNA pitch
(~10.5 bp)
Nucleosome
R (Ctrl) (n = 2)
R (JAKi) (n = 2)
G (Ctrl) (n = 2)
G (JAKi) (n = 2)
B
ATAC-seq
PC1 (62.33%)
PC2 (10.17%)
R (Ctrl)
R (JAKi)
G (Ctrl)
G (JAKi)
C
Integrated analysis (RNA-seq and ATAC-seq)
JAKi sensitive
JAKi insensitive
R (Ctrl)
R (JAKi)
G (Ctrl)
G (JAKi)
(n = 14)
APOL4
CALHM6
ETV7
FAM225A
GBP1P1
GBP5
IDO1
ISG20
KIAA0040
OPTN
PARP12
STAT1
TRAFD1
UBE2L6
(n = 10)
CCL3
CCL3L1
DCSTAMP
DENND1B
FGL2
GPAT3
GSAP
IKBKE
PSMA6
VRK2
z-score
-1.5
1.5

Figure S2. Differential regulation of chromatin accessibility and gene expression by JAK inhibition in THP-1-derived macrophages, **related to Figure 2. (A)** ATAC-seq fragment size distribution generated from nuclei shows chromatin-dependent periodicity, with a spatial frequency consistent with nucleosomes and a high-frequency periodicity consistent with the DNA helix pitch for fragments < 200 bp. Fragment length indicates the genomic distance between two Tn5 insertion sites. Density indicates the fraction of fragments with the indicated length. **(B)** PCA plot of ATAC-seq samples from THP-1-derived macrophages using total differential ACR (n = 38,950) identified by edgeR (FDR-adjusted $P < 0.05$, |Fold change| > 2) for all pairwise comparisons. Normalized tag count values of differential ACR were used. Each point represents an ATAC-seq sample. **(C)** Heatmap showing expression levels of DEG from THP-1-derived macrophages identified through integrated analysis of RNA-seq and ATAC-seq data. Representative genes from each category are listed on the right. The color scale represents z-scores.

Figure S3

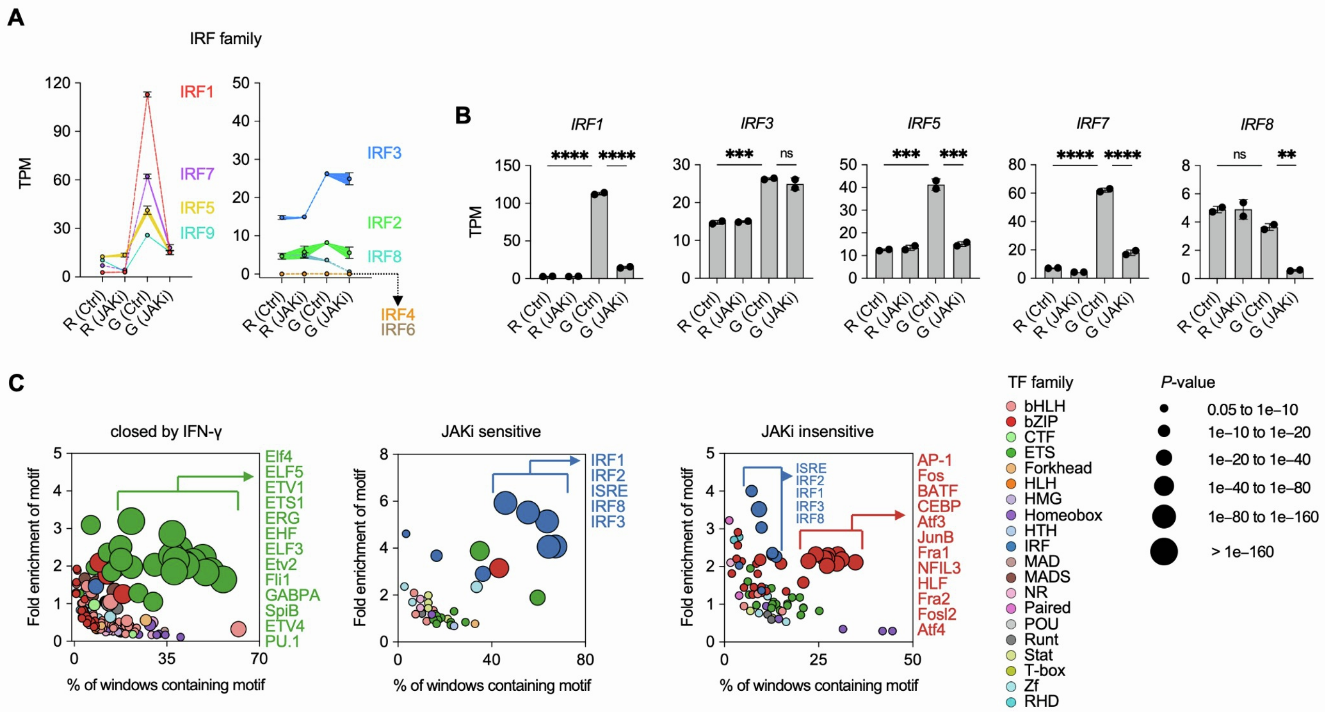

A
IRF family
TPM
IRF1
IRF7
IRF5
IRF9
IRF3
IRF2
IRF8
IRF4
IRF6
R (Ctrl)
R (JAKi)
G (Ctrl)
G (JAKi)
B
IRF1
IRF3
IRF5
IRF7
IRF8
ns
C
closed by IFN-γ
JAKi sensitive
JAKi insensitive
Fold enrichment of motif
% of windows containing motif
Elf4
ELF5
ETV1
ETS1
ERG
EHF
ELF3
Etv2
Fli1
GABPA
SpiB
ETV4
PU.1
IRF1
IRF2
ISRE
IRF8
IRF3
ISRE
IRF2
IRF1
IRF3
IRF8
AP-1
Fos
BATF
CEBP
Atf3
JunB
Fra1
NFIL3
HLF
Fra2
Fosl2
Atf4
TF family
bHLH
bZIP
CTF
ETS
Forkhead
HLH
HMG
Homeobox
HTH
IRF
MAD
MADS
NR
Paired
POU
Runt
Stat
T-box
Zf
RHD
P-value
0.05 to 1e−10
1e−10 to 1e−20
1e−20 to 1e−40
1e−40 to 1e−80
1e−80 to 1e−160
> 1e−160

Figure S3. Enrichment of specific motifs in JAKi-regulated ACR of THP-1-derived macrophages, related to Figure 3. (A) RNA-seq analysis showing expression patterns of IRF family members in THP-1 cells under different conditions. **(B)** Bar plots depicting the expression levels of selected IRF family members (*IRF1*, *IRF3*, *IRF5*, *IRF7*, and *IRF8*) under the indicated conditions. Data are presented as mean ± SD, with statistical significance determined by one-way ANOVA followed by Tukey's multiple comparisons test (ns, not significant; *P < 0.05; **P < 0.01; ***P < 0.001; ****P < 0.0001). **(C)** Bubble plot of TF motif enrichment analysis for each cluster from THP-1-derived macrophages. The motifs were identified by known motif analysis using HOMER. The most significantly enriched TF motif is shown on the right. Color range depicts different TF families and circle size refers to P-value significance.

Figure S4

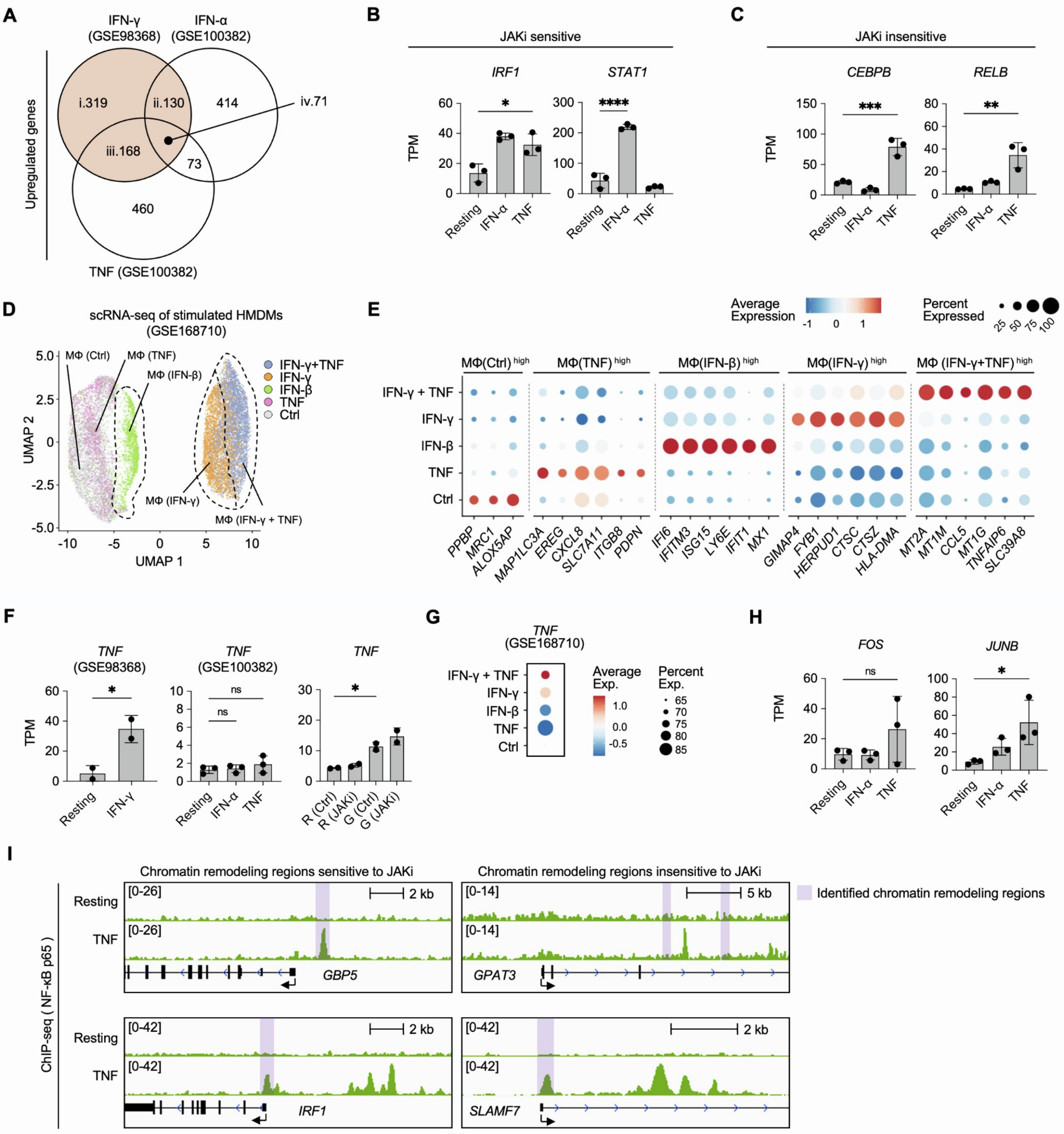

A
IFN-γ (GSE98368)
IFN-α (GSE100382)
TNF (GSE100382)
Upregulated genes
i.319
ii.130
iii.168
iv.71
414
73
460
B
JAKi sensitive
IRF1
STAT1
TPM
Resting
IFN-α
TNF
C
JAKi insensitive
CEBPB
RELB
D
scRNA-seq of stimulated HMDMs (GSE168710)
MΦ (Ctrl)
MΦ (TNF)
MΦ (IFN-β)
MΦ (IFN-γ)
MΦ (IFN-γ + TNF)
IFN-γ+TNF
IFN-γ
IFN-β
TNF
Ctrl
UMAP 1
UMAP 2
E
Average Expression
Percent Expressed
MΦ(Ctrl) high
MΦ(TNF) high
MΦ(IFN-β) high
MΦ(IFN-γ) high
MΦ (IFN-γ+TNF) high
PPBP
MRC1
ALOX5AP
MAP1LC3A
EREG
CXCL8
SLC7A11
ITGB8
PDPN
IFI6
IFITM3
ISG15
LY6E
IFIT1
MX1
GIMAP4
FYB1
HERPUD1
CTSC
CTSZ
HLA-DMA
MT2A
MT1M
CCL5
MT1G
TNFAIP6
SLC39A8
F
TNF (GSE98368)
TNF (GSE100382)
TNF
ns
R (Ctrl)
R (JAKi)
G (Ctrl)
G (JAKi)
G
TNF (GSE168710)
Average Exp.
Percent Exp.
H
FOS
JUNB
I
Chromatin remodeling regions sensitive to JAKi
Chromatin remodeling regions insensitive to JAKi
Identified chromatin remodeling regions
ChIP-seq (NF-κB p65)
Resting
TNF
[0-26]
[0-14]
[0-42]
2 kb
5 kb
GBP5
GPAT3
IRF1
SLAMF7

Figure S4. JAK inhibition differentially affects gene expression induced by IFN-γ, IFN-α, and TNF in human monocyte-derived macrophages (HMDM), related to Figure 4. (A) Venn diagram showing overlapping upregulated genes in HMDM stimulated with IFN-γ (GSE98368), IFN-α, and TNF (GSE100382). Numbers indicate upregulated gene counts. (**B and C)** Bar plots showing expression levels of JAKi-responsive genes defined from THP-1 analysis in different stimulation conditions. (B) JAKi-sensitive genes (*IRF1* and *STAT1*) and (C) JAKi-insensitive genes (*CEBPB* and *RELB*) expression levels in Resting, IFNα, and TNF conditions. **(D)** UMAP plot visualization of scRNA-seq data from stimulated HMDM (GSE168710). Dashed lines outline identified macrophage clusters under different stimulation conditions. **(E)** Dot plot matrix highlighting the expression patterns of select genes under various stimulation conditions in HMDM. The color intensity reflects the average gene expression, whereas the size of the dots represents the percentage of cells expressing the specific gene under the given condition. **(F)** Expression levels of *TNF* under various conditions in HMDM (GSE98368, GSE100382). **(G)** Dot plot showing expression levels of the *TNF* gene upon stimulation with IFN-γ, IFN-β, and TNF in HMDM (GSE168710). **(H)** Bar plots showing expression levels of AP-1 TF genes (*FOS* and *JUNB*) regulated by TNF in HMDM. **(I)** IGV tracks display NF-κB-p65 signals in HMDM under resting and TNF-stimulated conditions at JAKi-sensitive or JAKi-insensitive regions defined from THP-1 analysis (highlighted with colored boxes). Data are presented as mean ± SD, with statistical significance determined by one-way ANOVA followed by Tukey's multiple comparisons test (ns, not significant; $*P < 0.05$; $**P < 0.01$; $***P < 0.001$; $****P < 0.0001$).

Figure S5

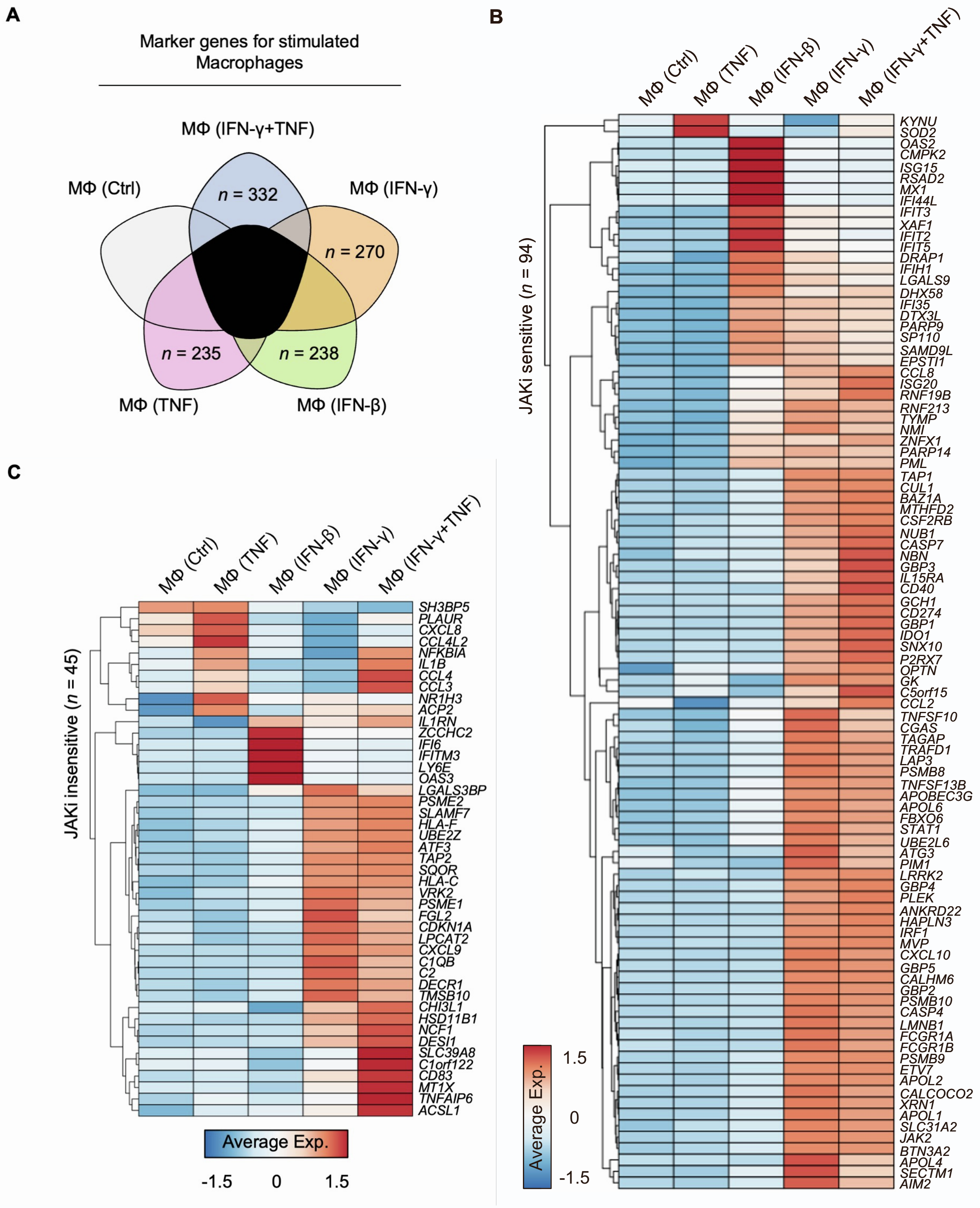
A
Marker genes for stimulated Macrophages
MΦ (IFN-γ+TNF)
MΦ (Ctrl)
MΦ (IFN-γ)
n = 332
n = 270
n = 235
n = 238
MΦ (TNF)
MΦ (IFN-β)
B
MΦ (Ctrl)
MΦ (TNF)
MΦ (IFN-β)
MΦ (IFN-γ)
MΦ (IFN-γ+TNF)
JAKi sensitive (n = 94)
KYNU
SOD2
OAS2
CMPK2
ISG15
RSAD2
MX1
IFI44L
IFIT3
XAF1
IFIT2
IFIT5
DRAP1
IFIH1
LGALS9
DHX58
IFI35
DTX3L
PARP9
SP110
SAMD9L
EPSTI1
CCL8
ISG20
RNF19B
RNF213
TYMP
NMI
ZNFX1
PARP14
PML
TAP1
CUL1
BAZ1A
MTHFD2
CSF2RB
NUB1
CASP7
NBN
GBP3
IL15RA
CD40
GCH1
CD274
GBP1
IDO1
SNX10
P2RX7
OPTN
GK
C5orf15
CCL2
TNFSF10
CGAS
TAGAP
TRAFD1
LAP3
PSMB8
TNFSF13B
APOBEC3G
APOL6
FBXO6
STAT1
UBE2L6
ATG3
PIM1
LRRK2
GBP4
PLEK
ANKRD22
HAPLN3
IRF1
MVP
CXCL10
GBP5
CALHM6
GBP2
PSMB10
CASP4
LMNB1
FCGR1A
FCGR1B
PSMB9
ETV7
APOL2
CALCOCO2
XRN1
APOL1
SLC31A2
JAK2
BTN3A2
APOL4
SECTM1
AIM2
Average Exp.
1.5
0
-1.5
C
MΦ (Ctrl)
MΦ (TNF)
MΦ (IFN-β)
MΦ (IFN-γ)
MΦ (IFN-γ+TNF)
JAKi insensitive (n = 45)
SH3BP5
PLAUR
CXCL8
CCL4L2
NFKBIA
IL1B
CCL4
CCL3
NR1H3
ACP2
IL1RN
ZCCHC2
IFI6
IFITM3
LY6E
OAS3
LGALS3BP
PSME2
SLAMF7
HLA-F
UBE2Z
ATF3
TAP2
SQOR
HLA-C
VRK2
PSME1
FGL2
CDKN1A
LPCAT2
CXCL9
C1QB
C2
DECR1
TMSB10
CHI3L1
HSD11B1
NCF1
DESI1
SLC39A8
C1orf122
CD83
MT1X
TNFAIP6
ACSL1
Average Exp.
-1.5
0
1.5

Figure S5. JAK inhibition differentially affects marker gene expression in human monocyte-derived macrophages (HMDM) under various stimulation conditions, **related to Figure 4. (A)** Venn diagram depicting the overlap of marker genes in HMDM under different stimulation conditions: control (Ctrl), IFN-γ, TNF, IFN-β, and combined IFN-γ+TNF. The numbers within each segment indicate the count of unique or overlapping marker genes among the conditions. **(B and C)** Hierarchical clustering heatmap of JAKi-responsive genes defined from THP-1 analysis showing (B) JAKi-sensitive and (C) JAKi-insensitive clusters across various stimulation conditions in HMDM. The color scale represents z-scores.

Figure S6

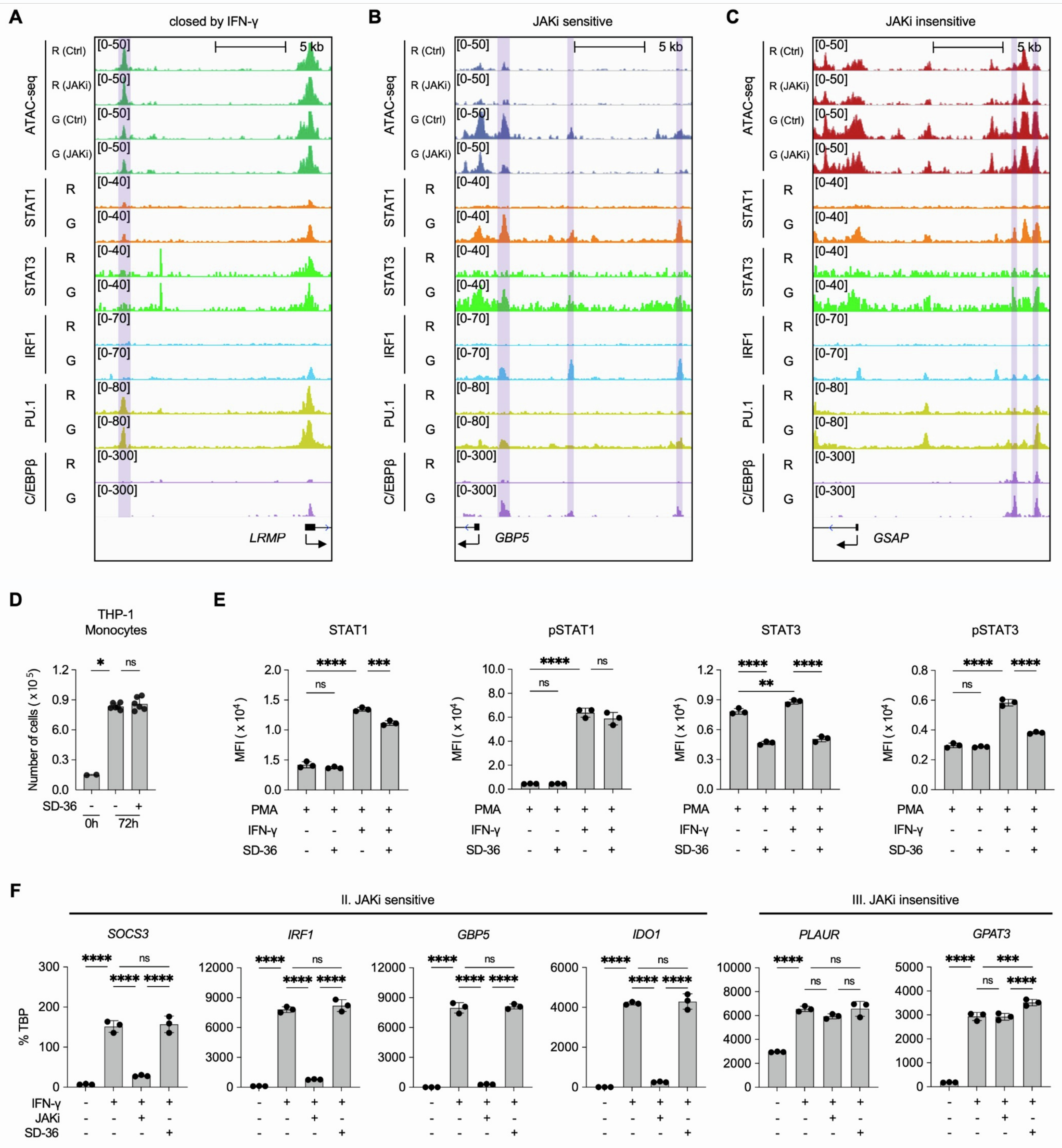
A
closed by IFN-γ
B
JAKi sensitive
C
JAKi insensitive
ATAC-seq
R (Ctrl)
R (JAKi)
G (Ctrl)
G (JAKi)
STAT1
STAT3
IRF1
PU.1
C/EBPβ
R
G
5 kb
LRMP
GBP5
GSAP
D
THP-1 Monocytes
Number of cells (x 10^5)
SD-36
0h
72h
E
STAT1
pSTAT1
STAT3
pSTAT3
MFI (x 10^4)
PMA
IFN-γ
SD-36
F
II. JAKi sensitive
III. JAKi insensitive
SOCS3
IRF1
GBP5
IDO1
PLAUR
GPAT3
% TBP
IFN-γ
JAKi
SD-36
ns

Figure S6. Variable TF occupancy in ACR affected by JAKi, **related to Figure 5. (A-C)** The IGV Genome Browser displays ATAC-seq signals from THP-1-derived macrophages and ChIP-seq signals (STAT1, STAT3, IRF1, PU.1 and C/EBPβ) from HMDM in the vicinities of indicated genes for chromatin remodeling regions. Chromatin regions that are "closed by IFN-γ", "JAKi-sensitive", and "JAKi-insensitive" identified from THP-1 ATAC-seq are highlighted with colored boxes. ATAC-seq, ChIP-seq and macrophage conditions are shown on the left (R, Resting macrophages; G, IFN-γ-primed macrophages). **(D)** Proliferation analysis of THP-1 monocytes treated with or without SD-36 (1 μM) at 0h and 72h. **(E)** Flow cytometric analysis of total and phosphorylated STAT1 and STAT3 levels in THP-1-derived macrophages. Cells were pre-treated with SD-36 for 24h prior to 3h stimulation with PMA and/or IFN-γ. Data are shown as mean fluorescence intensity (MFI). Three independent experiments were performed (n = 3). **(F)** RT-qPCR analysis of JAKi-sensitive (*SOCS3*, *IRF1*, *GBP5*, and *IDO1*) and JAKi-insensitive (*PLAUR* and *GPAT3*) genes. Cells were pre-treated with SD-36 for 24h, stimulated with PMA and/or IFN-γ for 24h, followed by 6h JAKi treatment. Three independent experiments were performed (n = 3). Data are presented as mean ± SD, with statistical significance determined by one-way ANOVA followed by Tukey's multiple comparisons test (ns, not significant; $^*P < 0.05$; $^{**}P < 0.01$; $^{***}P < 0.001$; $^{****}P < 0.0001$).

Figure S7

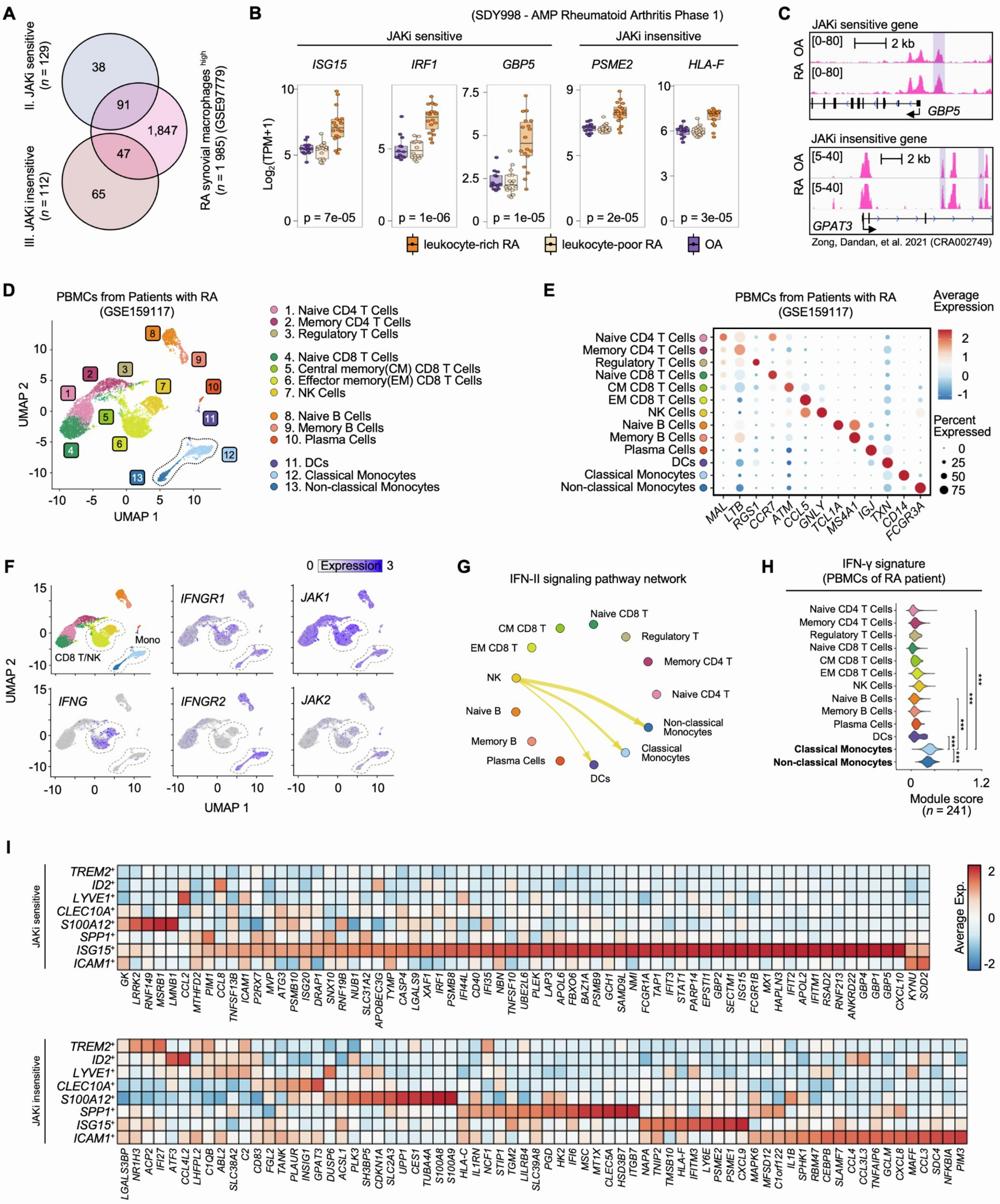

A
II. JAKi sensitive (n = 129)
III. JAKi insensitive (n = 112)
RA synovial macrophages high (n = 1 985) (GSE97779)
38
91
1,847
47
65
B
(SDY998 - AMP Rheumatoid Arthritis Phase 1)
JAKi sensitive
JAKi insensitive
ISG15
IRF1
GBP5
PSME2
HLA-F
Log2(TPM+1)
p = 7e-05
p = 1e-06
p = 1e-05
p = 2e-05
p = 3e-05
leukocyte-rich RA
leukocyte-poor RA
OA
C
JAKi sensitive gene
JAKi insensitive gene
GBP5
GPAT3
2 kb
Zong, Dandan, et al. 2021 (CRA002749)
D
PBMCs from Patients with RA (GSE159117)
1. Naive CD4 T Cells
2. Memory CD4 T Cells
3. Regulatory T Cells
4. Naive CD8 T Cells
5. Central memory(CM) CD8 T Cells
6. Effector memory(EM) CD8 T Cells
7. NK Cells
8. Naive B Cells
9. Memory B Cells
10. Plasma Cells
11. DCs
12. Classical Monocytes
13. Non-classical Monocytes
UMAP 1
UMAP 2
E
PBMCs from Patients with RA (GSE159117)
Average Expression
Percent Expressed
MAL LTB RGS1 CCR7 ATM CCL5 GNLY TCL1A MS4A1 IGJ TXN CD14 FCGR3A
F
Expression
IFNGR1
JAK1
IFNG
IFNGR2
JAK2
CD8 T/NK
Mono
G
IFN-II signaling pathway network
H
IFN-γ signature (PBMCs of RA patient)
Module score (n = 241)
I
JAKi sensitive
JAKi insensitive
TREM2+
ID2+
LYVE1+
CLEC10A+
S100A12+
SPP1+
ISG15+
ICAM1+
Average Exp.

Figure S7. Gene expression profile related to JAK inhibitor response in synovial macrophages from RA patients, **related to Figure 6. (A)** Venn diagram to identify genes associated with IFN-γ-primed macrophages in RA patients (GSE97779). **(B)** Expression profiling of JAKi-responsive genes defined from THP-1 analysis in synovial macrophages from OA and RA patients. **(C)** IGV genome browser displays ATAC-seq (CRA002749) signals for monocytes from OA and RA patients at chromatin regions defined as JAKi-sensitive or -insensitive in THP-1 cells (highlighted with colored boxes). **(D)** UMAP visualization of scRNA-seq data from PBMC of a RA patient (GSE159117). Dashed lines indicate identified monocyte clusters. **(E)** Dot plot of specific gene expression across different cell types from the RA patient's PBMC. Dot size indicates the percentage of cells expressing the gene; color gradient indicates average expression level. **(F)** Density plots for IFN-γ signaling components (*IFNG*, *IFNGR1*, *IFNGR2*, *JAK1*, and *JAK2*) in the UMAP space. Color gradient indicates expression level. **(G)** The inferred IFN-II signaling pathway network. The thickness of the line indicates the interaction strength. **(H)** IFN-γ signature module score analysis of scRNA-seq data obtained from PBMC of RA patients. Statistical significance of violin plots was assessed using the Wilcoxon test. **(I)** Heatmap shows expression of JAKi-responsive genes defined from THP-1 analysis across synovial macrophage subpopulations defined by various markers. The color scale represents z-scores.

Table S1. Primers for RT-qPCR, related to STAR Methods.

Target gene	Primer	Sequence (5' - 3')
TBP	Forward	CCC GAA ACG CCG AAT ATA ATC C
	Reverse	AAT CAG TGC CGT GGT TCG TG
LRMP	Forward	TCT GTG TGA AGA TGA CAA CCA GG
	Reverse	TGC TTT ACT AAC CCG GCT TTC
ITGB5	Forward	GGA AGT TCG GAA ACA GAG GGT
	Reverse	CTT TCG CCA GCC AAT CTT CTC
GBP5	Forward	TGC TAT CGA CCT ACT GCA CAA
	Reverse	GCA GGA TCT TCA ACC CTG TCA
IDO1	Forward	GCC AGC TTC GAG AAA GAG TTG
	Reverse	ATC CCA GAA CTA GAC GTG CAA
PLAUR	Forward	GAG CTA TCG GAC TGG CTT GAA
	Reverse	CGG CTT CGG GAA TAG GTG AC
GPAT3	Forward	GAA CTA CAC TGG TTG GGC AG
	Reverse	TCT GGG GTC TGT ACT GCT TG
CCL2	Forward	AAG CAG AAG TGG GTT CAG GA
	Reverse	GCT GCA GAT TCT TGG GTT GT
CXCL10	Forward	GAA TCG AAG GCC ATC AAG AA
	Reverse	GCT CCC CTC TGG TTT TAA GG
FCGR1A	Forward	AGC TGT GAA ACA AAG TTG CTC T
	Reverse	GGT CTT GCT GCC CAT GTA GA
CCR2	Forward	CCA CAT CTC GTT CTC GGT TTA TC
	Reverse	CAG GGA GCA CCG TAA TCA TAA TC
CD68	Forward	CTT CTC TCA TTC CCC TAT GGA CA
	Reverse	GAA GGA CAC ATT GTA CTC CAC C
ICAM1	Forward	TTG GGC ATA GAG ACC CCG TT
	Reverse	GCA CAT TGC TCA GTT CAT ACA CC
HIF1a	Forward	GAA CGT CGA AAA GAA AAG TCT CG
	Reverse	CCT TAT CAA GAT GCG AAC TCA CA
DUSP1	Forward	ACC ACC ACC GTG TTC AAC TTC
	Reverse	TGG GAG AGG TCG TAA TGG GG
HMOX1	Forward	CCA GTG CCA CCA AGT TCA AG
	Reverse	GAT GTT GAG CAG GAA CGC AG
IRF1	Forward	CTG TGC GAG TGT ACC GGA TG
	Reverse	ATC CCC ACA TGA CTT CCT CTT
SOCS3	Forward	CCT GCG CCT CAA GAC CTT C
	Reverse	GTC ACT GCG CTC CAG TAG AA

Table S2. Illumina/Nextera i5 common adapter and i7 index adapters, related to STAR Methods.

Adapter name	Sequence (5' - 3')
Ad1_noMX	AAT GAT ACG GCG ACC ACC GAG ATC TAC ACT CGT CGG CAG CGT CAG ATG TG
Ad2.1_TAAGGCGA	CAA GCA GAA GAC GGC ATA CGA GAT TCG CCT TAG TCT CGT GGG CTC GGA GAT GT
Ad2.2_CGTACTAG	CAA GCA GAA GAC GGC ATA CGA GAT CTA GTA CGG TCT CGT GGG CTC GGA GAT GT
Ad2.3_AGGCAGAA	CAA GCA GAA GAC GGC ATA CGA GAT TTC TGC CTG TCT CGT GGG CTC GGA GAT GT
Ad2.4_TCCTGAGC	CAA GCA GAA GAC GGC ATA CGA GAT GCT CAG GAG TCT CGT GGG CTC GGA GAT GT
Ad2.5_GGACTCCT	CAA GCA GAA GAC GGC ATA CGA GAT AGG AGT CCG TCT CGT GGG CTC GGA GAT GT
Ad2.6_TAGGCATG	CAA GCA GAA GAC GGC ATA CGA GAT CAT GCC TAG TCT CGT GGG CTC GGA GAT GT
Ad2.7_CTCTCTAC	CAA GCA GAA GAC GGC ATA CGA GAT GTA GAG AGG TCT CGT GGG CTC GGA GAT GT
Ad2.8_CAGAGAGG	CAA GCA GAA GAC GGC ATA CGA GAT CCT CTC TGG TCT CGT GGG CTC GGA GAT GT
